# An integrative single-cell multi-omics profiling of human pancreatic islets identifies T1D associated genes and regulatory signals

**DOI:** 10.21203/rs.3.rs-3343318/v1

**Published:** 2023-10-18

**Authors:** Zeping Zhao, Ricardo D’Oliveira Albanus, Henry Taylor, Xuming Tang, Yuling Han, Peter Orchard, Arushi Varshney, Tuo Zhang, Nandini Manickam, Mike Erdos, Narisu Narisu, Leland Taylor, Xiaxia Saavedra, Aaron Zhong, Bo Li, Ting Zhou, Ali Naji, Chengyang Liu, Francis Collins, Stephen CJ Parker, Shuibing Chen

**Affiliations:** 1 Department of Surgery, Weill Cornell Medicine, 1300 York Ave, New York, NY, 10065, USA; 2 Center for Genomic Health, Weill Cornell Medicine, 1300 York Ave, New York, NY 15 10065, USA; 3 Department of Computational Medicine and Bioinformatics, University of Michigan, Ann Arbor, MI, USA; 4 Center for Precision Health Research, National Human Genome Research Institute, National Institutes of Health, Bethesda, MD 20892, USA; 5 Stem Cell Research Facility, Memorial Sloan Kettering Cancer Center, 1275 York Avenue, New York, NY 10065, USA; 6 Genomic Resource Core Facility, Weill Cornell Medical College, NY 10065, USA; 7 Department of Surgery, University of Pennsylvania School of Medicine, Philadelphia, PA19104, USA.; 8 Department of Human Genetics, University of Michigan, Ann Arbor, MI, USA; 9 Department of Biostatistics, University of Michigan, Ann Arbor, MI, USA.

## Abstract

Genome wide association studies (GWAS) have identified over 100 signals associated with type 1 diabetes (T1D). However, translating any given T1D GWAS signal into mechanistic insights, including putative causal variants and the context (cell type and cell state) in which they function, has been limited. Here, we present a comprehensive multi-omic integrative analysis of single-cell/nucleus resolution profiles of gene expression and chromatin accessibility in healthy and autoantibody^+^ (AAB+) human islets, as well as islets under multiple T1D stimulatory conditions. We broadly nominate effector cell types for all T1D GWAS signals. We further nominated higher-resolution contexts, including effector cell types, regulatory elements, and genes for three independent T1D risk variants acting through islet cells within the pancreas at the *DLK1/MEG3, RASGRP1,* and *TOX* loci. Subsequently, we created isogenic gene knockouts *DLK1*^−/−^, *RASGRP1*^−/−^, and *TOX*^−/−^, and the corresponding regulatory region knockout, *RASGRP1*^*Δ*^, and *DLK1*^*Δ*^ hESCs. Loss of *RASGRP1* or *DLK1*, as well as knockout of the regulatory region of *RASGRP1* or *DLK1,* increased β cell apoptosis. Additionally, pancreatic β cells derived from isogenic hESCs carrying the risk allele of *rs3783355*^*A/A*^ exhibited increased β cell death. Finally, RNA-seq and ATAC-seq identified five genes upregulated in both *RASGRP1*^−/−^ and *DLK1*^−/−^ β-like cells, four of which are associated with T1D. Together, this work reports an integrative approach for combining single cell multi-omics, GWAS, and isogenic hESC-derived β-like cells to prioritize the T1D associated signals and their underlying context-specific cell types, genes, SNPs, and regulatory elements, to illuminate biological functions and molecular mechanisms.

## Introduction

Type 1 diabetes (T1D) is a complex autoimmune disease that represents 5–10% of all diagnosed diabetes cases ^1^. The primary manifestation of this disease is the targeting of pancreatic β cells by the immune system, likely mediated by T-cells, resulting in loss of β cells and insulin deficiency^2^. During T1D progression, the process of β cell destruction is marked by the production of autoantibodies (AAB^+^) to the β cell, occurs over many years and ultimately results in metabolic abnormalities first manifested as impaired glucose tolerance and then progressing to symptomatic hyperglycemia. The AAB+ patients cover both pre- and onset- T1D groups. The emergence of genotyping and imputation has significantly increased the power and accuracy for genome-wide association studies (GWAS) of T1D genetic risk^3,4^. It is widely accepted that immune cells are the primary mediators of T1D genetic risk^2^, which is supported by the strong genetic association of the major histocompatibility complex (MHC) in T1D GWAS^3,5^. However, mounting evidence suggests that other cell types, including pancreatic cells, also play critical roles in T1D etiology and genetic risk^3,4,6^. For example, one proposed mechanism for T1D risk variants acting through β cells is to modulate their susceptibility to immune-mediated apoptosis^7^. Two recent studies using functional genomics at the single-cell level have helped clarify some of the biology driving T1D genetic risk and contributing to T1D progression^4,8^. Both studies identified a role for non-immune cell types in the pancreas, particularly acinar and ductal cells, in mediating T1D genetic signals^4^ or contributing to T1D onset and progression^8^. Moreover, one of these studies reported that *cis*-regulatory elements active in β cells significantly overlapped with T1D GWAS variants^4^, indicating that β cells play a role in T1D genetic risk. However, there remains a lack of a systematic approach to prioritize and functionally characterize these genetic variants in human pancreatic β cells.

Several experiments have been performed to examine healthy and T1D human islets at single cell level. Early studies performed single cell RNA-seq (scRNA-seq) of T1D islets^8–10^. Additionally, combining T1D GWAS and chromatin accessibility profiling of pancreas and peripheral blood mononuclear cells using single-nucleus assay for transposase-accessible chromatin with sequencing (snATAC-seq) showed that risk variants for T1D were enriched in T cell open chromatin, as well as acinar and ductal cells^4^. Furthermore, recent studies using single-cell transcriptional profiling and in situ imaging mass cytometry identified a subset of exocrine ductal cells that acquires a signature of tolerogenic dendritic cells in an apparent attempt at immune suppression in T1D donors^8^. However, most of these previous studies have focused on the analysis of profiling data under basal conditions and without functional validation.

Human pluripotent stem cells (hPSCs), which includes human embryonic stem cells (hESCs) and induced pluripotent stem cells (iPSCs), have been used to model human diseases. Several studies have utilized hPSC-derived β-like cells to investigate pancreatic β cell dysfunction in diabetes. These studies have largely focused on maturity-onset diabetes of the young (MODY)^11–22^, and neonatal diabetes (NDM)^20,23,24^. In addition, isogenic hPSCs have been utilized to study genes implicated in T2D-implicated genes, such as *KCNQ1*, *KCNJ11, CDKAL1*, *GATA6*, *SIX2,* and *ABCC8*, in β cells^25^. Furthermore, isogenic *GLIS3−/−* hESCs showed the defective differentiation toward pancreatic β cells and the derived β cells showed increased cell death both *in vitro* and *in vivo*^26^. By combining high-throughput epigenome and single-cell nuclear sequencing with the hPSC-derived β cell platforms, researchers have been able to identify more diabetes risk genes or loci, such as *LAMA1*, *CRB2*, and *rs231361*^27^.

In this study, we performed single-cell resolution multi-omic integration of high-throughput molecular profiles of paired gene expression and chromatin accessibility of human islets from both healthy and AAB^+^ donors. Although T1D is a complex disease, researchers have used various *in vitro* conditions to mimic cytokine- or virus-induced beta cell damage. For example, a proinflammatory cytokine cocktail that includes interferon gamma (IFN-γ), interleukin 1b (IL-1β), and tumor necrosis factor α (TNF-α) has been used to stimulate inflammatory signaling^28^. Additionally, several viruses have been associated with T1D, including enteroviruses such as Coxsackievirus B (CVB), rotavirus, mumps virus, and cytomegalovirus^29^. Here, we used two *in vitro* models, including cytokine cocktail (TNF-α, IFN-g and IL-1β) treatment and coxsackievirus B4 virus (CVB4) infection^30,31^ to simulate the stressed environment β cells are exposed to during T1D. We used both AAB+ islets and cytokine- or CVB4-treated islets to characterize mechanisms of T1D genetic risk, focusing on identifying variants acting through islet endocrine cells. We prioritized three independent T1D risk variants acting through pancreatic islet endocrine cells at the *TOX*, *RASGRP1*, and *DLK1/MEG3* loci. Subsequently, we created isogenic gene knockout, region knockout, and SNP knockin hESC lines to characterize the biological functions of these genes, regulatory elements, and SNPs in human β cell survival.

## Results

### Integrative analysis of single cell gene expression and chromatin accessibility profiles of human islets.

**Extended Data Fig.1.**
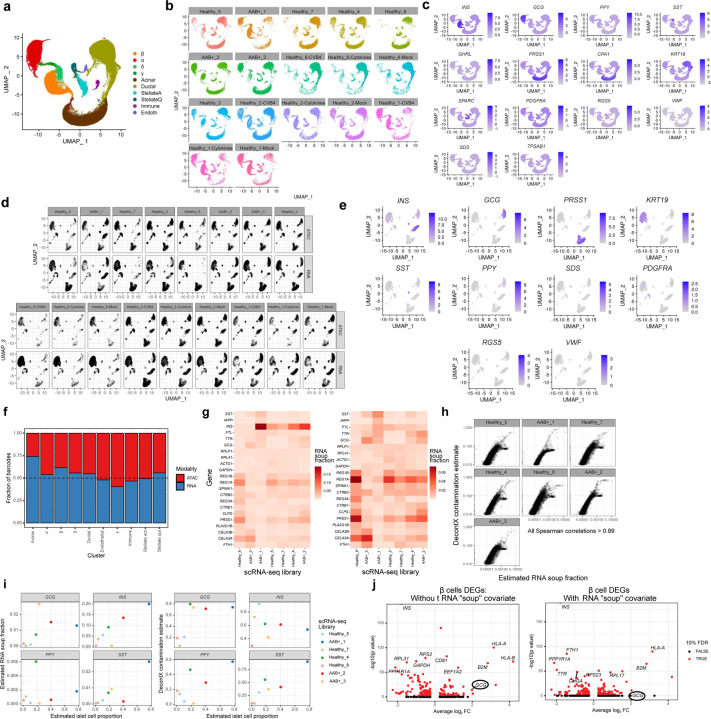
Functional genomics at the single-cell/nucleus QC and integration. **a**, UMAP representation of the first-pass scRNA-seq-only integration and clustering used as input for DecontX. **b**, UMAP representation split by samples. **c**, Marker gene expression in the first-pass scRNA-seq clustering. **d**, UMAP representation of integrated scRNA-seq and snATAC-seq data faceted by sample (columns) and modality (rows). **e**, Marker gene expression across clusters. **f**, Distribution of ATAC and RNA barcodes that passed QC for each cell type. **g**, Estimated ambient RNA (“RNA soup”) composition for a subset of scRNA-seq libraries, obtained by combining all barcodes with less than 10 UMIs (*i.e.* empty droplets). Right plot is the same as the left, but without *INS* for visibility. **h**, Agreement of the RNA contamination estimated by DecontX to ambient RNA fraction estimated directly from empty droplets. Clusters of off-diagonal genes correspond to ribosomal proteins. **i**, Comparison of ambient RNA fraction for each gene in the facets to the estimated islet proportion (fraction of barcodes assigned to the islet clusters) per library. **j**, DEGs in β cells between HPAP055 (AAB+) versus controls with and without a covariate accounting for ambient RNA. HAPAP055 has a higher fraction of α cells compared to the other samples, which leads to higher levels of *GCG* in the ambient RNA. This, in turn, leads to erroneous assignment of *GCG* as a DEG (left plot, black circle). This technical artifact is mitigated once we include the estimated alpha cells proportion in the sample as a proxy of ambient RNA (right plot, black circle). Differential expression performed using the negative binomial test from Seurat with number of UMIs, percent mitochondrial reads, age, and sex used as covariates.

**Fig. 1:**
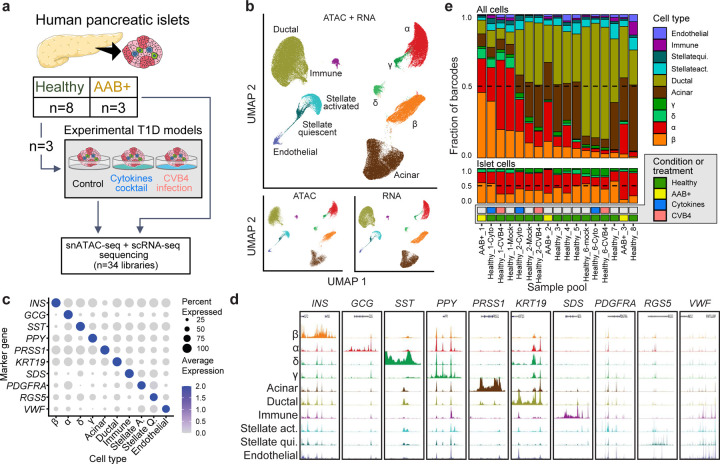
Integrative single cell multi-omics analysis of AAB+ human islets and islets cultured in T1D conditions. **a**, Experimental design for multi-omic library generation. **b,** Uniform Manifold Approximation and Projection (UMAP) representation of the fully integrated dataset. Bottom panel is the same data faceted by modality. **c**, scRNA average expression values for marker genes across the cell types identified via joint modality clustering. **d**, Normalized aggregate ATAC-seq signal tracks across marker genes for each cell type. **e**, Overview of the representation of all cell types (top), islet endocrine cell types (middle), and conditions (bottom) across the combined scRNA-seq and snATAC-seq libraries for each sample pool.

We performed single cell gene expression (scRNA-seq) and single nucleus chromatin accessibility (snATAC-seq) profiling on human pancreatic islets from healthy (n=8) and AAB^+^ donors (n=3). Considering the large donor-to-donor variation, we also performed scRNA-seq and snATAC-seq on islets from a subset of healthy donors (n=3) under cytokine stimulation (TNF-α, IFN-g and IL-1β) and CVB4 infection (Fig. 1a, Supplementary Table 1). After stringent quality control (QC; Methods), we profiled 121,272 cells (49,897 snATAC-seq nuclei and 71,375 scRNA-seq cells; Extended Data Fig. 1a–d, Supplementary Table 2). We performed joint clustering of the molecular profiles across samples and modalities (n=34 libraries) using Seurat^32^. We identified ten major distinct cell types based on the gene expression of known marker genes and the chromatin accessibility of their gene bodies (Fig. 1b–d, Extended Data Fig. 1e). The identified cell types represent the endocrine (α, β, δ, and γ cells), exocrine (acinar and ductal), stellate (activated and quiescent), endothelial, and immune lineages. Cell type representation ranged from 1.4% (immune) to 35% (ductal) of all cells. We profiled 41,569 islet endocrine cells and nuclei, corresponding to 34.3% of all profiled cells and nuclei. α cells were the most abundant endocrine cells (n=21,151), followed by β (n=15,577), δ (n=2,703), and γ cells (n=2,138). All cell types were well-represented across samples and modalities, and we did not identify any sample- or modality-specific clusters after QC (Fig. 1c, Extended Data Fig. 1d). Importantly, we observed during the initial QC steps that the ambient RNA contamination (RNA “soup”) was a source of technical variation across libraries and could lead to misinterpretation of results if not correctly accounted for (Methods, Extended Data Fig. 1g–j). Our findings are consistent with a recent study indicating that ambient RNAs can confound single-cell analyses.

### Transcriptional changes in experimental models of T1D recapitulate disrupted pathways in T1D.

**Extended Data Fig.2.**
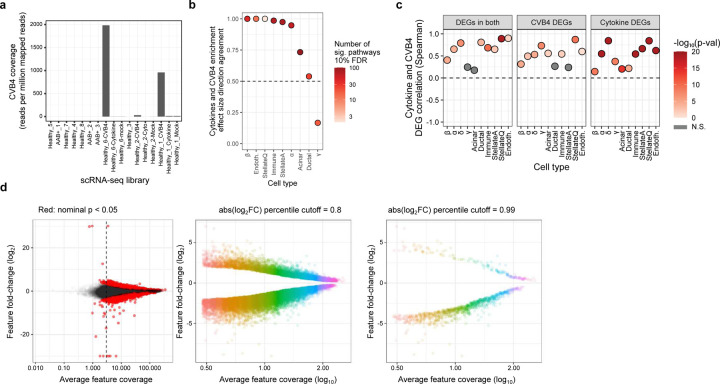
Differential analyses. **a**, Estimation of CVB4 infection efficiency per library using RNA-seq reads mapped to the CVB4 genome using a hybrid hg19-CVB4 genome. **b**, Pathway enrichments agreements between DEGs in cytokine stimulation and CVB4 infection across all cell types. **c**, DEG effect size correlation (Spearman) of nominally significant genes between cytokine stimulation and CVB4 infection. **d**, Example DAR significance calculation using effect sizes. Each color in the rainbow plots in the middle and right panels correspond to one of the 50 ATAC-seq signal bins.

Aiming to identify pathways and regulatory programs associated with T1D, we first performed differential expression analyses across disease states and experimental perturbations. We accounted for biological and technical covariates that could influence results to quantify differential expression across conditions accurately. After adjusting for technical variation, we detected 2,272 differentially expressed genes (DEGs) at 10% false discovery rate (FDR) across all cell types and conditions combined (ranging from 1 to 883 per cell type and condition, mean = 98; Supplementary Table 3). We observed lower transcriptional changes associated with CVB4 infection compared to cytokine stimulation in all cell types, which could be due to heterogeneity in the CVB4 infection efficiency across samples. Indeed, we observed differences in the number of detectable CVB4 mRNAs in each CVB4-treated sample (Extended Data Fig. 2a). This variability may explain why the CVB4 infection DEG effect sizes were generally smaller.

**Fig. 2:**
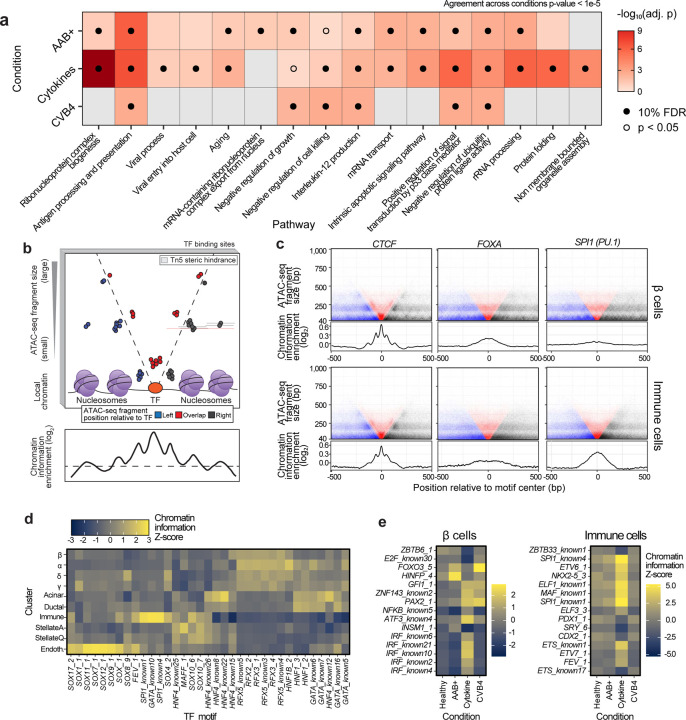
Transcriptomic changes associated with T1D and experimental models and TF regulatory landscape of pancreatic cell types. **a**, Significantly enriched pathways across AAB^+^ and experimental models. **b**, Chromatin information enrichment calculation overview (adapted from^34^). **c**, V-plots showing aggregate ATAC-seq fragment midpoints distribution around predicted bound sites for three TFs (top facets) and their associated chromatin information enrichment (bottom facets) in β cells and immune cells. **d**, Chromatin information Z-scores for a subset of TFs across all cell types indicate differential regulatory activity. **e**, Similar to D, but directly comparing across conditions for β and immune cells.

To determine if the experimental perturbations recapitulated functional aspects of progression towards T1D in pancreatic cells, we performed pathway enrichment analyses using the DEGs from disease state and perturbations. Due to the low number of AAB^+^ samples in our data, we opted to use the beta cells AAB^+^ DEGs reported by a recent, more well-powered study^33^. We found overall high concordance between the pathway enrichments in AAB^+^ compared to cytokine stimulation and CVB4 infection in β cells (Fig. 2a). These findings suggest cytokine stimulation and CVB4 infection affect similar pathways in β cells compared to the AAB^+^ cell state environment. In addition, we found a high agreement between the DEGs and pathways enriched in cytokine stimulation and CVB4 infection in the other cells types, suggesting that they elicit similar transcriptional changes (Extended Data Fig. 2b, c).

### Transcription factors regulating the epigenomic landscape of pancreatic cells.

To characterize the epigenomic landscape of the different pancreatic cell types, we used the BMO tool^34^ to predict bound transcription factor (TF) sites using a non-redundant collection of 540 motifs along with chromatin accessibility profiles in each cell type and calculated their corresponding chromatin information patterns. The observed chromatin information patterns around a given motif reflect the impact of specific TFs in organizing local chromatin architecture and establishing cell identity^34^ (Fig. 2d). We identified common and cell-type-specific TFs driving the epigenomic landscape for each cell type (Fig. 2e, f). The TFs CTCF, AP-1, and NFE2 consistently scored highest in chromatin information across cell types (Supplementary Table 4), likely reflecting their constitutive roles in chromatin organization^35,36^. On the other hand, a subset of TF families had a higher impact on chromatin organization in a cell-specific manner. These TF families include RFX and FOXA in endocrine cells, HNF in exocrine cells, and SPI1 (PU.1) in immune cells (Fig. 2f). The identified TF families have previously been characterized as cell fate determinants and play functional roles in their respective lineages^37–39^. This observation, therefore, underscores the specificity and rigor of our epigenomic analyses. Importantly, we observed changes in the underlying chromatin organization associated with a subset of TFs when comparing conditions (Fig. 2g). The IRF motif family was associated with increased chromatin organization in β cells under cytokine treatment, consistent with previous studies showing that cytokine stimulation induces IRF-1 activation in β cells and results in subsequent apoptosis^40,41^. In agreement with these results, we observed a significant up-regulation of *IRF1* in cytokine-treated β-cells compared to control (log_2_ fold-change = 5.37, adj. *p* = 1.12e-4; Supplementary Table 3) Similarly, cytokine treatment induced changes in chromatin organization at the SPI1, MAF, and ETS family TF motifs in immune cells, which are well-known mediators of cytokine response in these cells^42,43^. Notably, the chromatin organization changes in AAB+ cells were less pronounced than the environmental perturbations, which suggest that the experimental models of T1D associate with acute changes in cellular state occurring in a shorter time frame. Together, these results highlight the dynamic landscape of chromatin accessibility in pancreas cell types and identify TFs likely driving cellular identity and environmental response in islets and other pancreatic cells.

### Enrichment of T1D GWAS variants nominates cell types likely mediating T1D genetic risk.

**Fig. 3:**
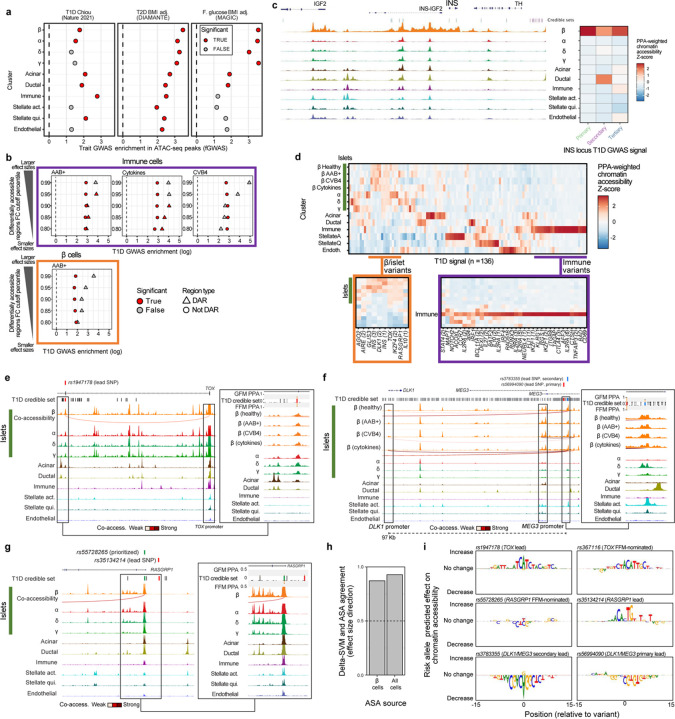
The regulatory landscape associated with T1D genetics in pancreatic cells. **a**, fGWAS enrichments for GWAS summary statistics of three traits in accessible chromatin regions from each cell type in our data. **b**, fGWAS enrichments for T1D summary statistics in immune and β cells across progressively stringent thresholds to identify differentially accessible regions (DARs) and their non-significant counterparts. **c**, Example of our PPA-weighted chromatin accessibility score strategy to identify cell types likely mediating three independent T1D GWAS signals at the INS locus. **d**, PPA-weighted chromatin accessibility scores across all T1D loci and cell types and candidate loci likely mediated by islet and immune cell types. **e-g**, T1D signals at the *TOX*, *DLK1/MEG3*, and *RASGRP1* loci. Left panels represent the broad locus overview, and the insets highlight the regions and variants of interest and their associated genetic and functional fine-mapping PPA values. For simplicity, only β-cell co-accessibility tracks are shown. **h**, Agreement between predicted and observed ATAC-seq allelic imbalance (allele-specific accessibility; ASA) in β cells and all cells using a predictive model trained in β cells. **i**, Predicted regulatory impact of T1D risk variants of interest in β cell chromatin accessibility using GkmExplain.

In order to investigate the mechanisms involved in T1D genetic risk, we used fGWAS^44^ to calculate the enrichment of the accessible chromatin of the different cell types captured by our snATAC-seq experiments using the summary statistics of a recent T1D GWAS^4^. As expected, we observed the highest T1D GWAS enrichment in the immune cluster (log enrichment = 2.78; Fig. 3a). The other significantly enriched cell types were acinar, quiescent stellate, β, ductal, and α cells (log enrichments ranging from 1.53 to 2.12). These results suggest that multiple pancreatic cell types, including islet endocrine cells, contribute to T1D genetic risk. These enrichments, however, represent the baseline (unperturbed) state of these cells and, therefore, may miss specific environmental contexts of T1D genetics. To contextualize these results, we tested the enrichment of accessible chromatin using the summary statistics of type 2 diabetes from the Diabetes Meta-Analysis of Trans-Ethnic association studies (DIAMANTE) Consortium^45^ and fasting glucose from the Meta-Analyses of Glucose and Insulin-related traits Consortium (MAGIC)^46^ GWAS studies. We observed the strongest enrichments for these two traits in accessible chromatin regions from β cells and other islet endocrine cell types (Fig. 3a), which is consistent with previous studies^45–48^, and highlights the quality of the underlying data and analyses in this current work.

We next investigated the context-specific roles of the studied cell types in T1D predisposition. To this end, we used fGWAS to calculate the enrichment of T1D GWAS summary statistics in differentially accessible regions (DARs) across disease states and experimental perturbations. Because of data sparseness and inflation of *p*-values associated with differential analyses in single-cell data^49^, we developed a stringent effect-size-based approach for detecting DARs in our snATAC-seq data (Extended Data Fig. 2d, Methods). As expected, DARs for AAB+ and cytokine treatment in immune cells were more highly enriched for T1D GWAS compared to non-DARs (Fig. 3b). In addition, the enrichment point estimates increased as we used more stringent DAR thresholds. This result is consistent with a substantial component of T1D genetic risk encoded by responsive elements in immune cells, such as the MHC locus^4^. We also observed a similar trend in DARs for CVB4 infection in immune cells, but it did not reach significance, potentially due to the difference in CVB4 infection efficiency across replicates (Extended Data Fig. 2a). Interestingly, we found that DARs in AAB+ β cells are more enriched for T1D GWAS than non-DARs. Similar to the previous results in immune cells, the enrichment point estimates for the β-cell DARs increased with more stringent DAR thresholds (Fig. 3b). This result suggests that the environmentally responsive regulatory elements in β cells also mediate T1D genetic risk and, therefore, indicate a role for islet endocrine cells in mediating T1D progression.

### Regulatory elements in β and other islet endocrine cells mediate T1D genetic risk.

Next, we aimed to understand regions and regulatory elements that are responsible for driving the observed T1D GWAS enrichments in pancreatic cells. To this end, we developed a novel approach to quantify the relative contributions of each cell type to T1D genetic risk and prioritize candidate cell types mediating genetic risk at a given locus. This approach is based on the cell-type-specific chromatin accessibility levels at each variant in a T1D genetic credible set, weighted by the posterior probability of association (PPA) of the variant (Methods). As a proof of concept, the three independent T1D GWAS signals at the *INS* locus were prioritized to act through β cells (Fig. 3c). A broader analysis of all 136 T1D GWAS signals showed that genetic risk is partitioned across all the cell types analyzed in this study (Fig. 3d). Immune cells contribute to most of the T1D genetic risk, as expected. However, we observed multiple signals prioritized to act through pancreatic endocrine (β, α, δ, γ), exocrine (acinar, ductal), stellate, and endothelial cells. Importantly, we identified several signals with β- or islet-specific accessibility, indicating that these genetic signals are likely mediated by islet endocrine cells in the pancreas. These islet endocrine loci include the three independent signals at the *INS* locus, the primary and secondary signals at *DLK1/MEG3*, and the signals at *TOX*, *RASGRP1*, and *GLIS3* (Fig. 3d).

We next attempted to prioritize T1D risk loci likely acting through β or other islet endocrine cells for functional validation. In addition to the PPA-weighted chromatin accessibility for each locus, we accounted for the number of variants in the 99% credible set (CS) and the PPA distribution across variants to nominate candidate loci where functional validation experiments were feasible. We prioritized loci with either a few variants in the 99% CS or loci where the PPA distribution was highly skewed towards a small number of variants. In addition, we used CICERO^50^ to calculate co-accessibility between variant-harboring regulatory elements and gene promoters to help identify candidate target genes. To further reduce the search space for candidate variants, we performed functional fine-mapping (FFM) with fGWAS using a joint model accounting for the chromatin accessibility peaks from cell types enriched for T1D GWAS (Supplementary Table 4; Methods). Using these criteria, we nominated the main signals at *TOX* (99% CS size = 28) and *RASGRP1* (99% CS size = 66) and the secondary signal *DLK1/MEG3* (10 variants with PPA > 0.01; 99% CS size = 2,053) as the most compelling candidate loci likely acting through β or islet endocrine cells (Fig. 3e, f).

At the *TOX* locus, our FFM analyses prioritized rs367116 and rs1947178, with the latter being the lead variant at the locus. The intronic β-cell regulatory element containing rs1947178 was co-accessible with the *TOX* promoter region, making *TOX* the candidate gene for this locus (Fig. 3e). At the *RASGRP1* locus, FFM prioritized rs55728265, which is in strong linkage disequilibrium (r^2^ = 0.93) with the lead variant, rs35134214. The regulatory element harboring rs55728265 overlaps the *RASGRP1* promoter region and was not co-accessible with any other promoter, making *RASGRP1* the candidate gene at this locus (Fig. 3f). The lead variant at this locus (*rs35134214*) did not overlap ATAC-seq peaks in any pancreatic cell types we observed, therefore highlighting the validity of using FFM approaches to prioritize the cell specificity of genetic signals. At the *DLK1/MEG3* locus, our FFM analyses prioritized the lead variant for the primary signal (*rs56994090*), despite this variant not overlapping any features used in the FFM model (Fig. 3g). We also prioritized the lead variant at the secondary signal at *DLK1/MEG3* (*rs3783355*; PPA = 0.56) because it had a 7-fold higher PPA compared to the second highest variant in the 99% CS (*rs10145648*; PPA = 0.08) and overlapped a highly accessible chromatin region in β, α, and ductal cells. Interestingly, we observed increased co-accessibility between the regulatory element harboring *rs3783355* and the *DLK1* and *MEG3* promoter regions in AAB^+^ or cytokine-stimulated β cells compared to healthy β cells (*MEG3*–*rs3783355* CICERO scores 0, 0.0001, and 0.013 for healthy, AAB^+^, and cytokine, respectively; *DLK1*–*rs3783355* CICERO scores 0.002, 0.0001, and 0.144 for healthy, AAB^+^, and cytokine, respectively). These results suggest that the regulatory element harboring *rs3783355* acts in a context-dependent manner to mediate T1D risk in pancreatic islet endocrine cells.

### T1D risk variants are predicted to disrupt islet endocrine cell regulatory elements.

**Extended Data Fig.3.**
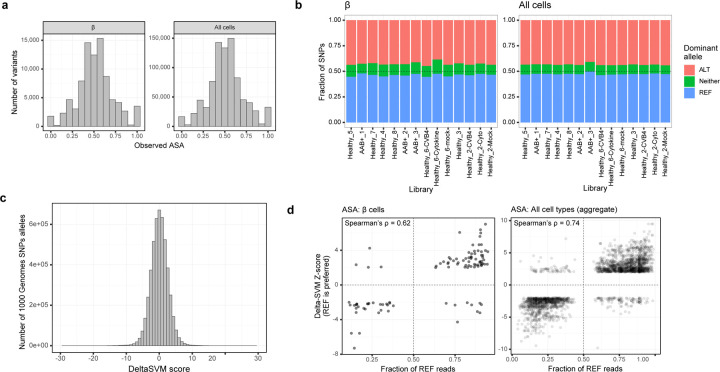
Predicting the regulatory impact of prioritized variants. **a**, **b**, Allele-specific accessibility (ASA) distribution in β cells and all cells for all heterozygous SNPs to estimate reference bias in WASP. **c**, DeltaSVM score distribution for all heterozygous SNPs. d, Effect size comparison between SNPs with significant ASA and DeltaSVM scores.

We next characterized the functional mechanisms through which the variants of interest at the *TOX*, *RASGRP1*, and *DLK1/MEG3* loci act to mediate T1D risk. We aimed to characterize the impact of the risk and non-risk alleles and because we had genotype information for 10 of the donors, we calculated the cell type-specific ATAC-seq allelic bias at each heterozygous SNP with enough coverage (Extended Data Fig. 3a, b). In parallel, we trained a predictive model of sequence features associated with chromatin accessibility in β cells using LS-GKM and DeltaSVM^51,52^ to predict β-cell allelic effects associated with any base-pair change in the genome (Methods; Extended Data Fig. 3c, d). We used the observed allelic bias to validate our predictive model. The predicted allelic effects from the model were highly concordant (87.1% effect direction agreement; binomial test *p* = 1.36e-99) with the observed allelic effects (ATAC-seq allelic bias) at heterozygous SNPs, indicating that the model correctly captured allelic regulatory changes associated with increased chromatin accessibility in β cells (Fig. 3h). The predictions from the model trained in β cells had a higher agreement with the observed allelic effects calculated using the entire dataset (92.6% effect direction agreement; binomial test *p* < 1e-323), which we attribute to increased statistical power to detect allelic bias effects when combining data across all cell types. Alternatively, this also can be interpreted as the model trained in β cells also capturing sequence features associated with chromatin accessibility more broadly.

To further gain information from our predictive model, we applied GkmExplain^53^ to the variants of interest and predicted the regulatory effects associated with each allele within the entire sequence context around the variants (Fig. 3i). At the *TOX* locus, the risk allele at the lead variant, rs1947178 (risk = A; non-risk = G), was predicted to increase chromatin accessibility. The predicted impact for the risk allele at rs1947178 was also higher than that of the other nominated SNP, rs367116 (risk = C; non-risk = T). At the *RASGRP1* locus, the FFM-nominated SNP, rs55728265 (risk = T; non-risk = C), was predicted to decrease accessibility. Finally, at the *DLK1/MEG3* locus, we predicted stronger effects in chromatin accessibility associated with the risk allele at the secondary signal lead variant, rs3783355 (risk = G; non-risk = A) compared to the lead variant at the primary signal (rs56994090; risk = T, non-risk = C). Consistent with the predicted effects in dysregulating chromatin accessibility, we identified multiple predicted bound TF motifs overlapping these risk variants, including PAX4 and HNF4 (*RASGRP1*), ITGB2, and ZBTB6 (*DLK1/MEG3*), and CPHX (*TOX*) (Supplementary Table 6). Together, these results implicate *rs1947178* (*TOX*), *rs55728265* (*RASGRP1*), and *rs3783355* (*DLK1/MEG3*) as likely causal variants mediating T1D genetic risk through islet cell types.

### Isogenic *DLK1*^−/−^ and *RASGRP1*^−/−^ hESC -derived pancreatic β-like cells show increased apoptosis.

**Extended Data Fig.4.**
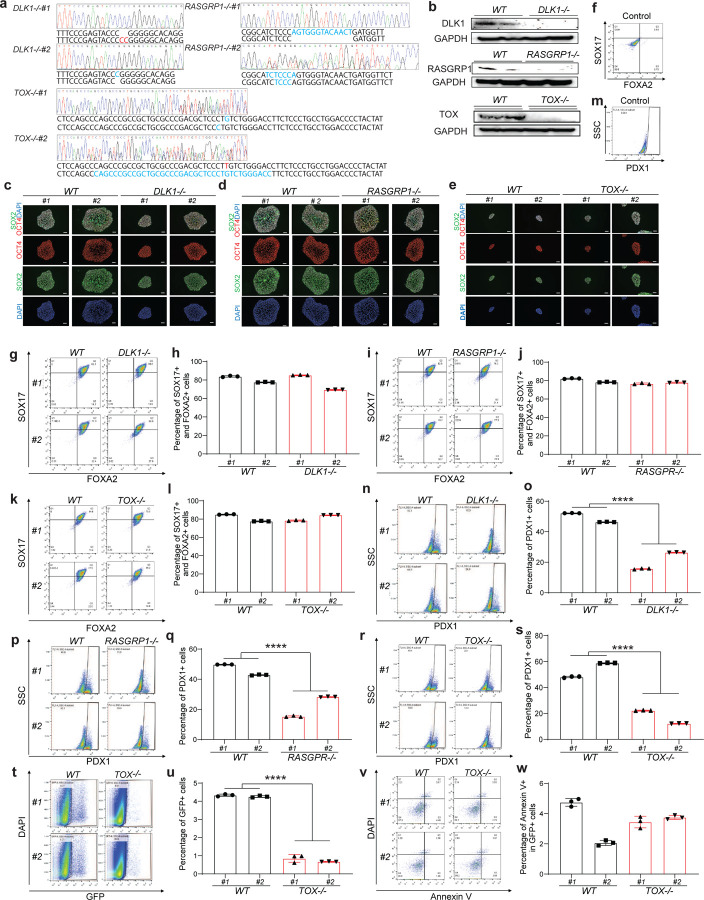
Characterization and stepwise differentiation of *DLK1*^−/−^, *RASGRP1*^−/−^*, TOX*^−/−^ and *WT* hESCs. **a,** DNA sequencing of *DLK1*^−/−^, *RASGRP1*^−/−^ and *TOX*^−/−^ hESCs. Blue color highlighted the deleted nucleotides. Red color highlighted the inserted nucleotides. **b,** Western blotting analysis of DLK1, RASGRP1 or TOX expression level in their wildtype (*WT*) and *DLK1*^−/−^, *RASGRP1*^−/−^ and *TOX*^−/−^ hESC-derived cells. **c-e**, Immunostaining of pluripotency markers of *DLK1*^−/−^ (**c**), *RASGRP1*^−/−^ (**d**)*, TOX*^−/−^ (**e**) and their *WT* hESCs. Scale bar=100 μm. **f-h**, Isotype control (**f**), representative flow cytometry analysis (**g**) and the quantification (**h**) of the percentage of SOX17^+^ and FOXA2^+^ cells in *WT* and *DLK1*^−/−^ hESC-derived cells. N = 3 biological replicates. **i, j**, Representative flow cytometry analysis (**i**) and the quantification (**j**) of the percentage of SOX17^+^ and FOXA2^+^ cells in *WT* and *RASGRP1*^−/−^ hESC-derived cells. N = 3 biological replicates. **k, l**, Representative flow cytometry analysis (**k**) and the quantification (**l**) of the percentage of SOX17^+^ and FOXA2^+^ cells in *WT* and *TOX*^−/−^ hESC-derived cells. N = 3 biological replicates. **m-o**, Isotype control (**m**), representative flow cytometry analysis (**n**) and the quantification (**o**) of the percentage of PDX1^+^ cells in *WT* and *DLK1*^−/−^ hESC-derived cells. N = 3 biological replicates. **p, q**, Representative flow cytometry analysis (**p**) and the quantification (**q**) of the percentage of PDX1^+^ cells in *WT* and *RASGRP1*^−/−^ hESC-derived cells. N = 3 biological replicates. **r, s**, Representative flow cytometry analysis (**r**) and the quantification (**s**) of the percentage of PDX1^+^ cells in *WT* and *TOX*^−/−^ hESC-derived cells. N = 3 biological replicates. **t**, **u,** Representative flow cytometry analysis (**t**) and the quantification (**u**) of the percentage of GFP^+^ cells in *WT* and *TOX*^−/−^ hESC-derived cells. N=3 biological replicates. **v**, **w,** Representative flow cytometry analysis (**v**) and the quantification of the percentage of Annexin V^+^DAPI^−^ cells (**w**) in *WT* and *TOX*^−/−^ hESC-derived *INS*-GFP^+^ cells under non-treated condition. N=3 biological replicates. P values were ****P < 0.0001. The center value is “mean”. Error bar is SEM.

To confirm the genetic variants and loci identified from our single cell RNA-seq analysis, we firstly created *DLK1*^−/−^*, RASGRP1*^−/−^ and *TOX*^−/−^ hESC cells based on *INS*^*GFP/W*^ MEL1 cells, which enables us to purify INS-GFP^+^ cells. In brief, *INS*^*GFP/W*^ MEL1 cells were electroporated with a vector expressing CAS9, sgRNAs targeting *DLK1, RASGRP1* and *TOX* exons (Supplemental Table 7). Following puromycin selection and sub-cloning, multiple independent clones were expanded. Two clones of each isogenic line (#1 and #2) were used for further analysis to control for potential clone-to-clone variation. Successful knockout of all three genes were confirmed with genomic DNA sequencing (Extended Data Fig.4a). The deletion of DLK1, RASGRP1, and TOX were reaffirmed in *DLK1*^−/−^*, RASGRP1*^−/−^ and *TOX*^−/−^ hESCs by western blot (Extended Data Fig.4b). Immunocytochemistry staining confirms the typical colony morphology and the expression of pluripotency markers of all *WT*, *DLK1*^−/−^*, RASGRP1*^−/−^ and *TOX*^−/−^ hESC lines (Extended Data Fig.4c–e).

**Fig. 4.**
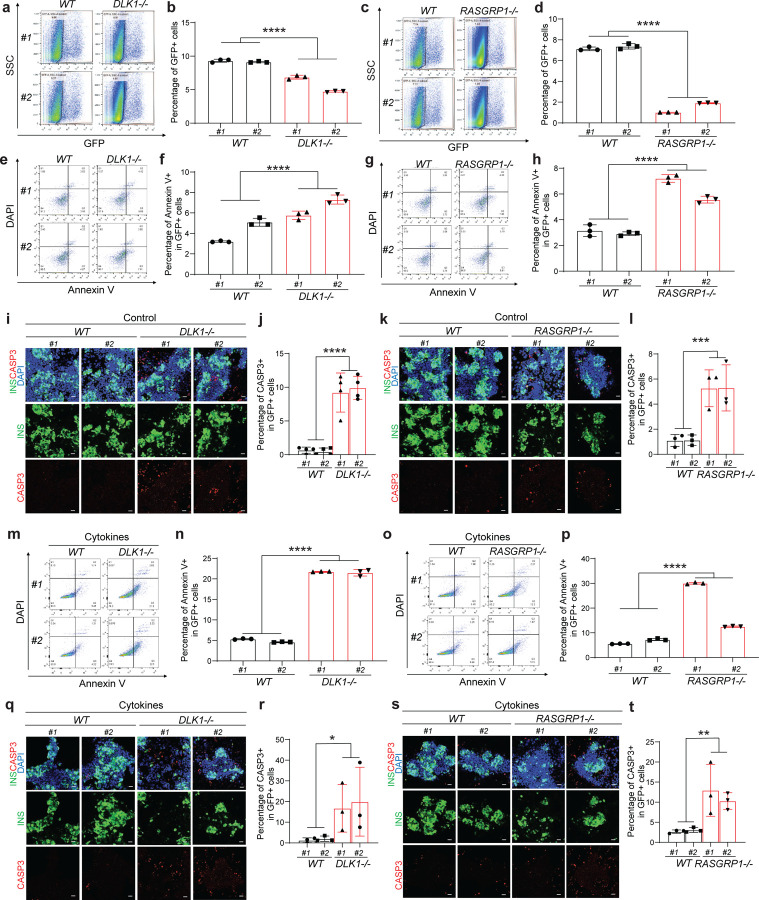
Isogenic *DLK1*^−/−^ and *RASGRP1*^−/−^ hESC-derived β cells show increased apoptosis. **a**, **b,** Representative flow cytometry analysis (**a**) and the quantification (**b**) of the percentage of GFP^+^ cells in *WT* and *DLK1*^−/−^ hESC-derived cells. N=3 biological replicates. **c**, **d,** Representative flow cytometry analysis (**c**) and the quantification (**d**) of the percentage of GFP^+^ cells in *WT* and *RASGRP1*^−/−^ hESC-derived cells. N=3 biological replicates. **e**, **f,** Representative flow cytometry analysis (**e**) and the quantification of the percentage of Annexin V^+^DAPI^−^ cells (**f**) in *WT* and *DLK1*^−/−^ hESC-derived *INS*-GFP^+^ cells under regular culture condition. N=3 biological replicates. **g**, **h,** Representative flow cytometry analysis (**g**) and the quantification of the percentage of Annexin V^+^DAPI^−^ cells (**h**) in *WT* and *RASGRP1*^−/−^ hESC-derived *INS*-GFP^+^ cells under regular culture condition. N=3 biological replicates. **i**, **j,** Representative images (**i**) and the quantification of the percentage of CASP3^+^ cells (**j**) in *WT* and *DLK1*^−/−^ hESC-derived *INS*-GFP^+^ cells under regular culture condition. N=3–5 biological replicates. **k**, **l,** Representative images (**k**) and the quantification of the percentage of CASP3^+^ cells (**l**) in *WT* and *RASGRP1*^−/−^ hESC-derived *INS*-GFP^+^ cells under regular culture condition. N=3 biological replicates. **m**, **n,** Representative flow cytometry analysis (**m**) and the quantification of the percentage of Annexin V^+^DAPI^−^ cells (**n**) in *WT* and *DLK1*^−/−^ hESC-derived *INS*-GFP^+^ cells under cytokines-treated condition. N=3 biological replicates. **o**, **p,** Representative flow cytometry analysis (**o**) and the quantification of the percentage of Annexin V^+^DAPI^−^ cells (**p**) in *WT* and *RASGRP1*^−/−^ hESC-derived *INS*-GFP^+^ cells under cytokines-treated condition. N=3 biological replicates. **q**, **r,** Representative images (**q**) and the quantification of the percentage of CASP3^+^ cells (**r**) in *WT* and *DLK1*^−/−^ hESC-derived *INS*-GFP^+^ cells under cytokines-treated condition. N=3 biological replicates. **s**, **t,** Representative images (**s**) and the quantification of the percentage of CASP3^+^ cells (**t**) in *WT* and *RASGRP1*^−/−^ hESC-derived *INS*-GFP^+^ cells under cytokines-treated condition. N=3 biological replicates. Scale bar = 40 μm. CASP3: cleaved caspase-3. P values were *P < 0.05, **P < 0.01, ***P < 0.001, ****P < 0.0001. The center value is “mean”. Error bar is SEM.

To systemic exam the role of *DLK1, RASGRP1,* and *TOX* in human pancreatic β cell generation, isogenic *WT*, *DLK1*^−/−^, *RASGRP1*^−/−^, and *TOX*^−/−^ hESC lines were differentiated into pancreatic β-like cells following our previous published protocol^5^. Flow cytometry analysis using antibodies against definitive endoderm (DE) markers, SOX17^+^/FOXA2^+^, confirmed *DLK1*^−/−^*, RASGRP1*^−/−^ and *TOX*^−/−^ hESC lines do not show impaired differentiation efficiency at definitive endoderm stage (Extended Data Fig. 4f–l). However, flow cytometry analysis with antibodies against pancreatic progenitor (PP) marker, PDX1, revealed that loss of *DLK1, RASGRP1,* or *TOX* led to the impaired PP differentiation (*WT#1*: 52±0.1%, *WT#2*: 46±0.1%, *DLK1*^−/−^*#1*: 16±0.2%, *DLK1*^−/−^*#2*: 26±0.1%, *P* < 0.0001; *WT#1*: 50±0.1%, *WT#2*: 43±0.2%, *RASGRP1*^−/−^*#1*: 15±0.3%, *RASGRP1*^−/−^*#2*: 28±0.1%, *P* < 0.0001; *WT#1*: 48±0.3%, *WT#2*: 59±0.1%, *TOX*^−/−^*#1*: 22±0.1%, *TOX*^−/−^*#2*: 12±0.1%, *P* < 0.0001) (Extended Data Fig. 4m–s). Furthermore, flow cytometry analysis demonstrated that deletion of any gene led to a lower percentage of INS-GFP^+^ cells (*WT#1*: 9.3±0.1%, *WT#2*: 9.1±0.1%, *DLK1*^−/−^*#1*: 6.9±0.2%, *DLK1*^−/−^*#2*: 4.7±0.1%, *P* < 0.0001; *WT#1*: 7.1±0.1%, *WT#2*: 7.4±0.2%, *RASGRP1*^−/−^*#1*: 1.0±0.1%, *RASGRP1*^−/−^*#2*: 1.9±0.1%, *P* < 0.0001; *WT#1*: 4.3±0.1%, *WT#2*: 4.3±0.1%, *TOX*^−/−^*#1*: 0.9±0.1%, *TOX*^−/−^*#2*: 0.7±0.1%, *P* < 0.0001) (Fig. 4a–d and Extended Data Fig. 4t–u). Collectively, these findings suggest that DLK1, RASGRP1, and TOX play crucial roles in regulating PP generation and pancreatic β cell differentiation.

To further investigate whether the decreased percentage of INS-GFP^+^ in *DLK1*^−/−^*, RASGRP1*^−/−^ and *TOX*^−/−^ hESC-derived pancreatic β-like cell is solely due to impaired differentiation of PP and pancreatic β cells or a result of both impaired differentiation and β apoptosis, we examined the early apoptotic rate of *DLK1*^−/−^*, RASGRP1*^−/−^ and *TOX*^−/−^ β-like cells (the percentage of Annexin V^+^DAPI^−^ cells in INS-GFP^+^ cells). Under the regular culture condition, the early apoptotic rate of *DLK1*^−/−^ *and RASGRP1*^−/−^ INS-GFP^+^ cells is significantly higher than that of *WT* INS-GFP^+^ cells (*WT#1*: 3.2±0.1%, *WT#2*: 5.1±0.2%, *DLK1*^−/−^*#1*: 5.8±0.2%, *DLK1*^−/−^*#2*: 7.3±0.3%, *P* < 0.0001; *WT#1*: 3.2±0.3%, *WT#2*: 2.9±0.1%, *RASGRP1*^−/−^*#1*: 7.2±0.2%, *RASGRP1*^−/−^*#2*: 5.6±0.2%, *P* < 0.0001) (Fig. 4e–h). Immunostaining further validates the increased apoptosis (the percentage of cleaved caspase 3/CASP3^+^ cells in INS^+^ cells) in *DLK1*^−/−^ *and RASGRP1*^−/−^ hESC-derived INS-GFP^+^ cells (Fig. 4i–l). However, we did not observe the similar phenotype in *TOX*^−/−^ INS-GFP^+^ cells (Extended Data Fig.4v, w). To further study the role of *DLK1* and *RASGRP1* in regulating β cell apoptosis in T1D condition, we also examined the early apoptotic rate of *DLK1*^−/−^ and *RASGRP1*^−/−^ β-like cells treated with cytokine cocktail (TNFα, IL1β and IFNγ). *DLK1*^−/−^ and *RASGRP1*^−/−^ INS-GFP^+^ cells showed significantly increased apoptotic rate than *WT* INS-GFP^+^ cells (*WT#1*: 5.3±0.1%, *WT#2*: 4.6±0.1%, *DLK1*^−/−^*#1*: 22±0.1%, *DLK1*^−/−^*#2*: 22±0.5%, *P* < 0.0001; *WT#1*: 5.5±0.1%, *WT#2*: 7.2±0.2%, *RASGRP1*^−/−^*#1*: 30±0.2%, *RASGRP1*^−/−^*#2*: 13±0.1%, *P* < 0.0001) upon cytokine cocktail treatment (Fig. 4m–p). Immunocytochemistry staining further confirmed the increased apoptotic rate in *DLK1*^−/−^ *and RASGRP1*^−/−^ β-like cells in cytokines treatment conditions (Fig. 4q–4t). Taken together, this suggested that the deletion of *TOX* mainly impairs pancreatic β cell differentiation, whereas the deletion of *DLK1* and *RASGRP1* might contribute to T1D progression through both impairing pancreatic β cell differentiation and elevating apoptosis.

### Deletion of *DLK1* and *RASGRP1* regulatory region led to increased β cell apoptosis.

**Extended Data Fig.5.**
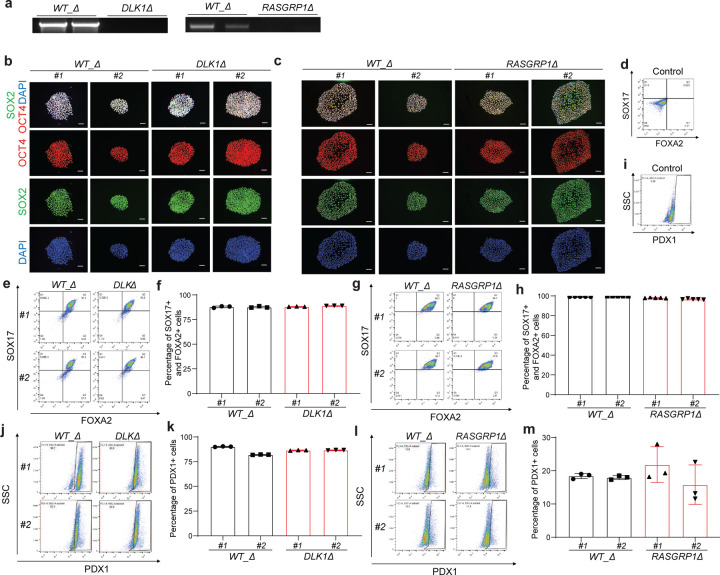
Characterization and stepwise differentiation of *DLK1*^*Δ*^, *RASGRP1*^*Δ*^ and their *WT_Δ* hESCs. **a**, PCR verification of *DLK1*^*Δ*^, *RASGRP1*^*Δ*^ and their *WT_Δ* hESCs. **b, c**, Immunostaining of pluripotency markers of *DLK1*^*Δ*^ (**b**), *RASGRP1*^*Δ*^ (**c**), and their *WT* hESCs. Scale bar=100 μm. **d-f**, Isotype control (**d**), representative flow cytometry analysis (**e**) and the quantification (**f**) of the percentage of SOX17^+^ and FOXA2^+^ cells in *WT_Δ* and *DLK1*^*Δ*^ hESC-derived cells. N = 3 biological replicates. **g, h**, Representative flow cytometry analysis (**g**) and the quantification (**h**) of the percentage of SOX17^+^ and FOXA2^+^ cells in *WT_Δ* and *RASGRP1*^*Δ*^ hESC-derived cells. N = 5 biological replicates. **i**-**k**, Isotype control (**i**), representative flow cytometry analysis (**j**) and the quantification (**k**) of the percentage of PDX1^+^ cells in *WT* and *DLK1*^*Δ*^ hESC-derived cells. N = 3 biological replicates. **l, m**, Representative flow cytometry analysis (**l**) and the quantification (**m**) of the percentage of PDX1^+^ cells in *WT* and *RASGRP1*^*Δ*^ hESC-derived cells. N = 3 biological replicates. The center value is “mean”. Error bar is SEM.

**Fig. 5.**
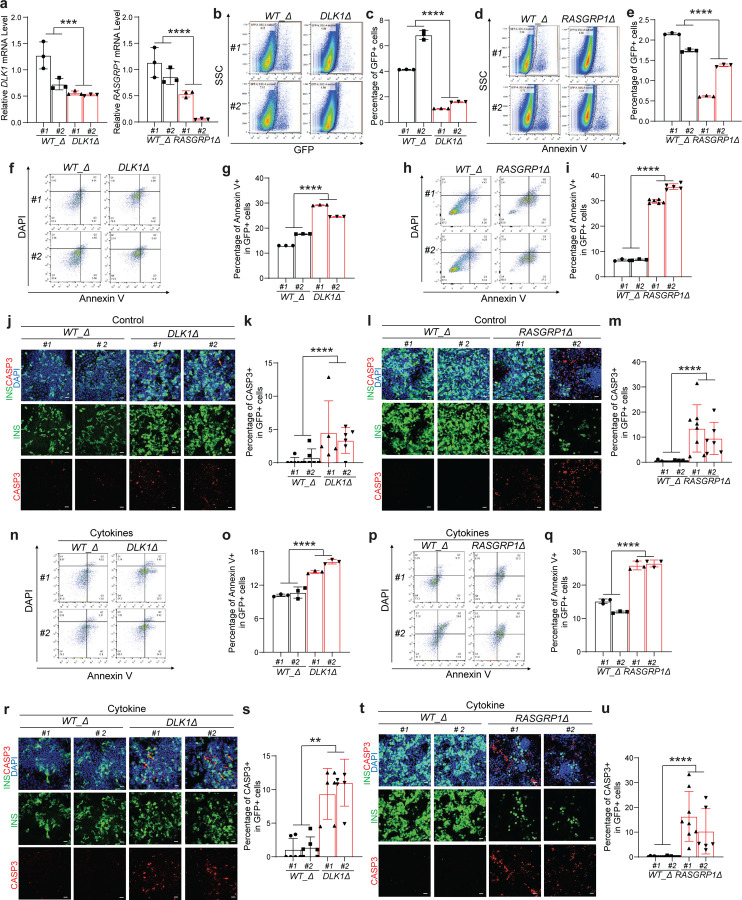
Knockout of the regulatory region of *DLK1* or *RASGRP1* causes the increased β cell apoptosis. **a,** qRT-PCR analysis of *DLK1* or *RASGRP1* mRNA level in isogenic *DLK1*^*Δ*^, *RASGRP1*^*Δ*^ and their *WT_Δ* hESC-derived *INS*-GFP^+^ cells. **b**, **c,** Representative flow cytometry analysis (**b**) and the quantification (**c**) of the percentage of GFP^+^ cells in *WT_Δ* and *DLK1*^*Δ*^ hESC-derived cells. N=3 biological replicates. **d**, **e,** Representative flow cytometry analysis (**d**) and the quantification (**e**) of the percentage of GFP^+^ cells in *WT_Δ* and *RASGRP1*^*Δ*^ hESC-derived cells. N=3 biological replicates. **f**, **g,** Representative flow cytometry analysis (**f**) and the quantification of the percentage of Annexin V^+^DAPI^−^ cells (**g**) in *WT_Δ* and *DLK1*^*Δ*^ hESC-derived *INS*-GFP^+^ cells under non-treated condition. N=3 biological replicates. **h**, **i,** Representative flow cytometry analysis (**h**) and the quantification of the percentage of Annexin V^+^DAPI^−^ cells (**i**) in *WT_Δ* and *RASGRP1*^*Δ*^ hESC-derived *INS*-GFP^+^ cells under non-treated condition. N=3–6 biological replicates. **j**, **k,** Representative images (**j**) and the quantification of the percentage of CASP3^+^ cells (**k**) in *WT_Δ* and *DLK1*^*Δ*^ hESC-derived *INS*-GFP^+^ cells under non-treated condition. N=5 or 6 biological replicates. **l**, **m,** Representative images (**l**) and the quantification of the percentage of CASP3^+^ cells (**m**) in *WT_Δ* and *RASGRP1*^*Δ*^ hESC-derived *INS*-GFP^+^ cells under non-treated condition. N=6 or 8 biological replicates. **n**, **o,** Representative flow cytometry analysis (**n**) and the quantification of the percentage of Annexin V^+^DAPI^−^ cells (**o**) in *WT_Δ* and *DLK1*^*Δ*^ hESC-derived *INS*-GFP^+^ cells under cytokines-treated condition. N=3 biological replicates. **p**, **q,** Representative flow cytometry analysis (**p**) and the quantification of the percentage of Annexin V^+^DAPI^−^ cells (**q**) in *WT_Δ* and *RASGRP1*^*Δ*^ hESC-derived *INS*-GFP^+^ cells under cytokines-treated condition. N=3 biological replicates. **r**, **s,** Representative images (**r**) and the quantification of the percentage of CASP3^+^ cells (**s**) in *WT_Δ* and *DLK1*^*Δ*^ hESC-derived *INS*-GFP^+^ cells under cytokines-treated condition. N=5 or 6 biological replicates. **t**, **u,** Representative images (**t**) and the quantification of the percentage of CASP3^+^ cells (**u**) in *WT_Δ* and *RASGRP1*^*Δ*^ hESC-derived *INS*-GFP^+^ cells under cytokines-treated condition. N=6 or 8 biological replicates. Scale bar = 40 μm. CASP3: cleaved caspase-3. P values were **P < 0.01, ****P < 0.0001. The center value is “mean”. Error bar is SEM.

To determine the impact of SNP contain regulator regions, we knocked out the regulatory region (chr14:101306805–101311032) containing *rs3783355* related to *DLK1* and region (chr15:38854520–38859072) containing *rs55728265* related to *RASGRP1* based on the analysis with human GWAS data mentioned above. We created *DLK1*^*Δ*^ and *RASGRP1*^*Δ*^ hESC cells based on *INS*^*GFP/W*^ MEL1 cells using two sgRNAs targeting the upstream and downstream of the regulatory regions of *DLK1* or *RASGRP1*, respectively (Supplemental Table 7). Successful knockout of the regulatory regions of *DLK1* or *RASGRP1* were confirmed with PCR (Extended Data Fig.5a and Supplemental Table 8). Immunocytochemistry staining validates the colony morphology and the expression of hESC pluripotency markers in *DLK1*^*Δ*^, *RASGRP1*^*Δ*^ and their *WT*(*WT_Δ*) clones (Extended Data Fig.5b, c). qRT-PCR confirmed the decreased expression of *DLK1* or *RASGRP1* in *DLK1*^*Δ*^ or *RASGRP1*^*Δ*^ hESC-derived INS-GFP^+^ cells (Fig. 5a and Supplemental Table 9). Flow cytometry analysis with antibodies against stepwise differentiation markers were applied to exam the differentiation efficiency of isogenic *DLK1*^*Δ*^, *RASGRP1*^*Δ*^ and the corresponding *WT_Δ* hESCs. *DLK1*^*Δ*^ and *RASGRP1*^*Δ*^ hESCs do not show impaired differentiation efficiency at DE (Extended Data Fig.5d–h) and PP stages (Extended Data Fig.5i–m), suggesting that deletion of the regulatory region showed milder phenotype than gene knockout. Flow cytometry analysis showed a significantly lower percentage of INS-GFP^+^ cells in *DLK1*^*Δ*^ and *RASGRP1*^*Δ*^ hESC-derived population than those of *WT_Δ* hESC-derived population (*WT_Δ#1*: 4.1±0.1%, *WT_Δ#2*: 6.8±0.2%, *DLK1*^*Δ*^*#1*: 1.1±0.1%, *DLK1*^*Δ*^*#2:* 1.6±0.1%, *P* < 0.0001; *WT_Δ#1*: 2.2±0.1%, *WT_Δ#2*: 1.7±0.1%, *RASGRP1*^*Δ*^*#1*: 0.6±0.1%, *RASGRP1*^*Δ*^*#2:* 1.4±0.1%, *P* < 0.0001) (Fig. 5b–e). Consistently with *DLK1*^−/−^ *and RASGRP1*^−/−^ β-like cells, both flow cytometry analysis (*WT_Δ#1*: 10±0.1%, *WT_Δ#2*: 11±0.6%, *DLK1*^*Δ*^*#1*: 14±0.1%, *DLK1*^*Δ*^*#2:* 16±0.2%, *P* < 0.0001; *WT_Δ#1*: 6.6±0.2%, *WT_Δ#2*: 6.8±0.1%, *RASGRP1*^*Δ*^*#1*: 30±0.4%, *RASGRP1*^*Δ*^*#2:* 36±0.6%, *P* < 0.0001, Fig. 5f–i) and immunostaining (Fig. 5j–m) showed that the early apoptotic rate of *DLK1*^*Δ*^ and *RASGRP1*^*Δ*^ *INS*-GFP^+^ cells is significantly higher than their *WT_Δ* INS-GFP^+^ cells under regulator culture condition. Upon the cytokines-treated condition, the apoptotic rates are higher in *DLK1*^*Δ*^ and *RASGRP1*^*Δ*^ INS-GFP^+^ cells than *WT_Δ* INS-GFP^+^ cells as indicated by both flow cytometry (*WT_Δ#1*: 13±0.1%, *WT_Δ#2*: 18±0.1%, *DLK1*^*Δ*^*#1*: 29±0.1%, *DLK1*^*Δ*^*#2:* 25±0.1%, *P* < 0.0001; *WT_Δ#1*: 15±0.4%, *WT_Δ#2*: 12±0.2%, *RASGRP1*^*Δ*^*#1*: 26±0.8%, *RASGRP1*^*Δ*^*#2:* 27±0.6%, *P* < 0.0001, Fig. 5n–q) and immunostaining (Fig. 5r–u).

### *rs3783355*^*G>A*^ mutation led to increased β cell apoptosis.

**Extended Data Fig.6.**
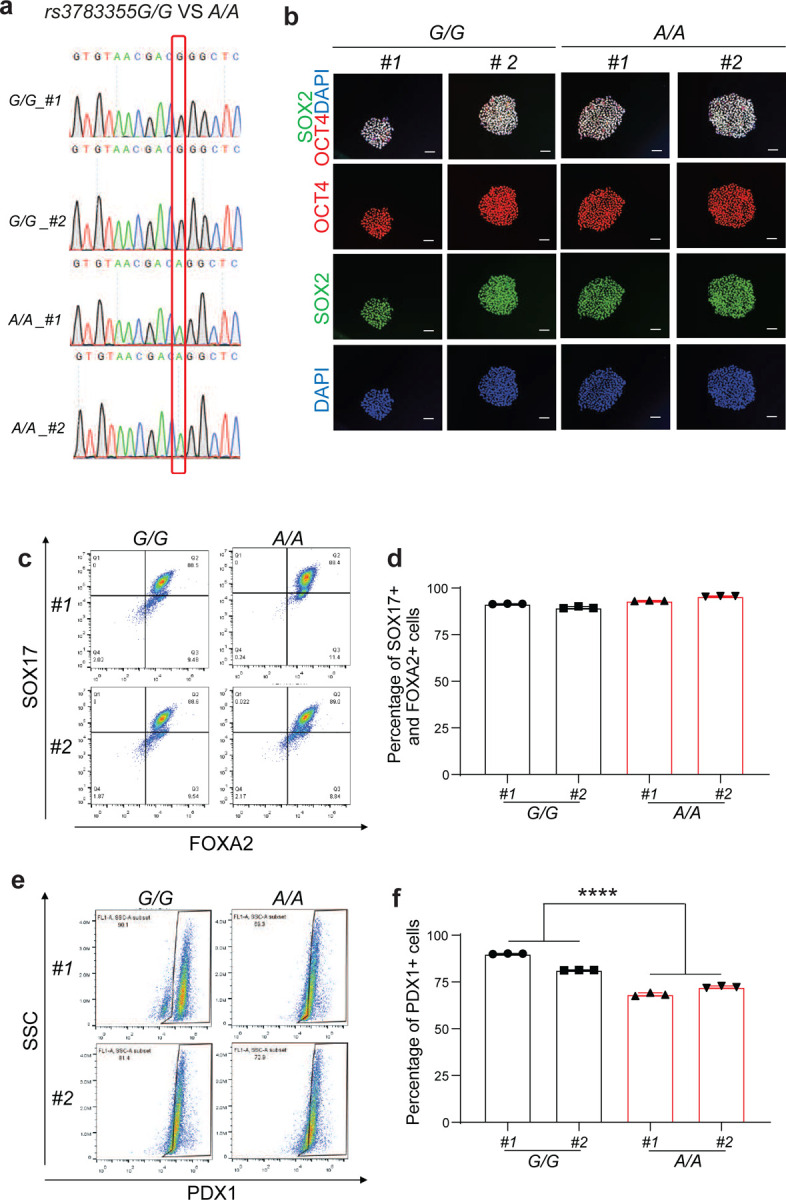
Characterization and stepwise differentiation of *rs3783355*^*G/G*^ and *rs3783355*^*A/A*^ hESCs. **a**, DNA sequencing of *rs3783355*^*G/G*^ and *rs3783355*^*A/A*^ isogenic hESC clones. **b**, Immunostaining of pluripotency markers of *rs3783355*^*G/G*^ and *rs3783355*^*A/A*^ isogenic hESC clones. Scale bar=100 μm. **c, d,** Representative flow cytometry analysis (**c**) and the quantification (**d**) of the percentage of SOX17^+^ and FOXA2^+^ cells in *rs3783355*^*G/G*^ and *rs3783355*^*A/A*^ hESC-derived cells. N = 3 biological replicates. **e**, **f,** Representative flow cytometry analysis (**e**) and the quantification (**f**) of the percentage of PDX1^+^ cells in *rs3783355*^*G/G*^ and *rs3783355*^*A/A*^ hESC-derived cells. N = 3 biological replicates. P values were ****P < 0.0001. The center value is “mean”. Error bar is SEM.

**Fig. 6.**
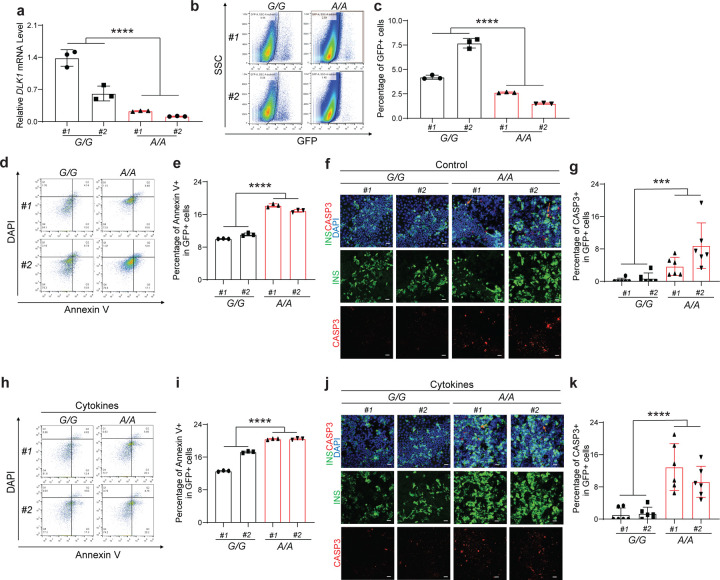
*rs3783355G>A* mutation results in the increased β cell apoptosis. **a,** qRT-PCR analysis of *DLK1* mRNA level in isogenic *rs3783355*^*G/G*^ and *rs3783355*^*A/A*^ hESC-derived *INS*-GFP^+^ cells. **b**, **c,** Representative flow cytometry analysis (**b**) and the quantification (**c**) of the percentage of GFP^+^ cells in *rs3783355*^*G/G*^ and *rs3783355*^*A/A*^ hESC-derived cells. N=3 biological replicates. **d**, **e,** Representative flow cytometry analysis (**d**) and the quantification of the percentage of Annexin V^+^DAPI^−^ cells (**e**) in *rs3783355*^*G/G*^ and *rs3783355*^*A/A*^ hESC-derived *INS*-GFP^+^ cells under regular culture condition. N=3 biological replicates. **f**, **g,** Representative images (**f**) and the quantification of the percentage of CASP3^+^ cells (**g**) in *rs3783355*^*G/G*^ and *rs3783355*^*A/A*^ hESC-derived *INS*-GFP^+^ cells under regular culture condition. N=6 biological replicates. **h**, **i,** Representative flow cytometry analysis (**h**) and the quantification of the percentage of Annexin V^+^DAPI^−^ cells (**i**) in *rs3783355*^*G/G*^ and *rs3783355*^*A/A*^ hESC-derived *INS*-GFP^+^ cells under cytokines-treated condition. N=3 biological replicates. **j**, **k,** Representative images (**j**) and the quantification of the percentage of CASP3^+^ cells (**k**) in *rs3783355*^*G/G*^ and *rs3783355*^*A/A*^ hESC-derived *INS*-GFP^+^ cells under cytokines-treated condition. N=6 biological replicates. Scale bar = 40 μm. CASP3: cleaved caspase-3. P values were ***P < 0.001, ****P < 0.0001. The center value is “mean”. Error bar is SEM.

Next, we tried to apply isogenic knockin hESC lines to examine the SNPs identified in the GWAS studies (Fig. 3). We successfully created isogenic *rs3783355*^*G/G*^ and *rs3783355*^*A/A*^ hESC lines, but failed in *rs3783355* isogenic hESC lines due to the high GC content of the region containing *rs55728265*. Similar to the description above, *INS*^*GFP/W*^ MEL1 cells were electroporated with a vector expressing CAS9, an sgRNA targeting the *rs3783355* site and a DNA-repairment template with *rs3783355*^*A/A*^ (Supplemental Table 7). After selection, sub-cloning and expansion, two *rs3783355*^*G/G*^ and *rs3783355*^*A/A*^ clones (#1 and #2) were used for further analysis. Successful knockin of risk A allele was confirmed with genomic DNA sequencing (Extended Data Fig.6a and Supplemental Table 8). Immunocytochemistry staining confirms the colony morphology and expression of pluripotency markers in *rs3783355*^*G/G*^ *and rs3783355*^*A/A*^ hESC lines (Extended Data Fig.6b). qRT-PCR analysis proved that *rs3783355 G>A* mutation caused the decrease of *DLK1* expression in the purified INS-GPF^+^ pancreatic β-like cells (17.3±2.6%, Fig. 6a and Supplemental Table 9). Flow cytometry analysis showed that *rs3783355*^*A/A*^ hESCs exhibited similar DE differentiation as *rs3783355*^*G/G*^ hESCs (Extended Data Fig.6c, d), but decreased PP differentiation compared to *rs3783355*^*G/G*^ hESCs (*G/G#1*: 90±0.1%, *G/G#2*: 81±0.1%, *A/A#1*: 68±0.5%, *A/A#2*: 72±0.3%, *P* < 0.0001) (Extended Data Fig.6e, f). Flow cytometry analysis also confirmed a significantly lower percentage of INS-GFP^+^ cells in *rs3783355*^*A/A*^ than that of *rs3783355*^*G/G*^ hESC-derived cells (*G/G#1*: 4.2±0.1%, *G/G#2*: 7.7±0.3%, *A/A#1*: 2.6±0.1%, *A/A#2*: 1.5±0.1%, *P* < 0.0001) (Fig. 6b, c). Both flow cytometry analysis (*G/G#1*: 10±0.1%, *G/G#2*: 11±0.2%, *A/A#1*: 18±0.2%, *A/A#2*: 17±0.2%, *P* < 0.0001, Fig. 6d, e) and immunostaining (Fig. 6f, g) showed that the apoptotic rate of *rs3783355*^*A/A*^ INS-GFP^+^ cells is significantly higher than *rs3783355*^*G/G*^ INS-GFP^+^ cells in regular culture condition. Consistently, in cytokines-treated condition, the apoptotic rate of *rs3783355*^*A/A*^ INS-GFP^+^ cells is significantly higher than *rs3783355*^*G/G*^ INS-GFP^+^ cells as indicated by both flow cytometry and (*G/G#1*: 13±0.1%, *G/G#2*: 17±0.1%, *A/A#1*: 20±0.1%, *A/A#2*: 20±0.1%, *P* < 0.0001, Fig. 6h, i) and immunostaining (Fig. 6j–k).

### Absence of *DLK1* and *RASGRP1* induce pancreatic β cell apoptosis through different pathways but also share common targets.

**Extended Data Fig.7.**
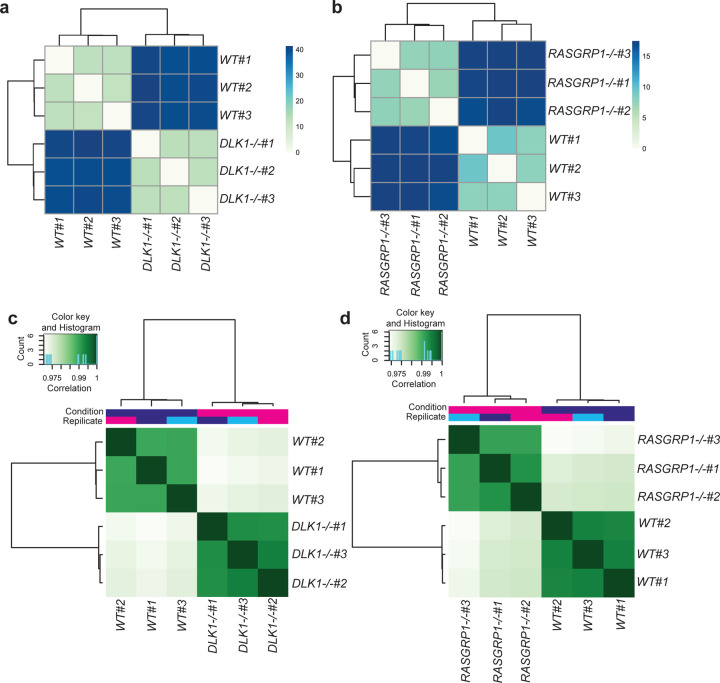
Cluster analysis of samples of RNA-seq and ATAC-seq. **a,** Diagram of RNA-seq result of *WT versus DLK1*^−/−^ *INS*-GFP^+^ cells. **b,** Diagram of ATAC-seq result of *WT versus DLK1*^−/−^ *INS*-GFP^+^ cells. **c,** Diagram of RNA-seq result of *WT versus RASGRP1*^−/−^ *INS*-GFP^+^ cells. **d,** Diagram of ATAC-seq result of *WT versus RASGRP1*^−/−^ *INS*-GFP^+^ cells.

**Fig. 7.**
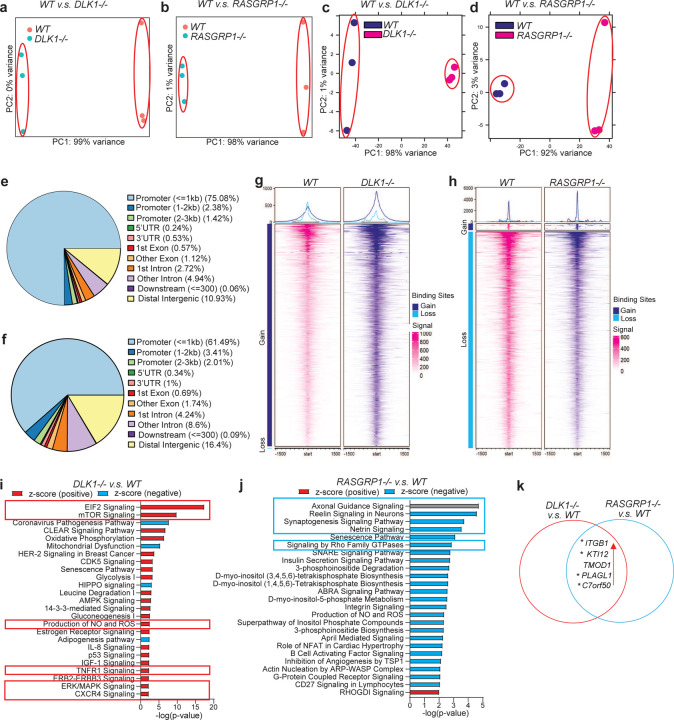
*DLK1* and *RASGRP1* induce β cell apoptosis through different pathways but share common target genes. **a,** PCA plot of RNA-seq result of *WT* versus *DLK1*^−/−^ *INS*-GFP^+^ cells. **b,** PCA plot of ATAC-seq result of *WT* versus *DLK1*^−/−^ *INS*-GFP^+^ cells. **c,** Pie chart of ATAC-seq result of *WT* versus *DLK1*^−/−^ *INS*-GFP^+^ cells. **d,** Profile heatmap plot showing the enrichment of Gain/Loss sites in the *DLK1*^−/−^ versus *WT INS*-GFP^+^ cells. **e,** IPA pathway analysis of upregulated or downregulated pathways in the *DLK1*^−/−^ versus *WT INS*-GFP^+^ cells. **f,** PCA plot of RNA-seq result of *WT* versus *RASGRP1*^−/−^ *INS*-GFP^+^ cells. **g,** PCA plot of ATAC-seq result of *WT* versus *RASGRP1*^−/−^ *INS*-GFP^+^ cells. Pie chart of ATAC-seq result of *WT* versus *RASGRP1*^−/−^ *INS*-GFP^+^ cells. **h,** Pie chart of ATAC-seq result of *WT* versus *RASGRP1*^−/−^ *INS*-GFP^+^ cells. **i,** Profile heatmap plot showing the enrichment of Gain/Loss sites in the *RASGRP1*^−/−^ versus *WT INS*-GFP^+^ cells. **j,** IPA pathway analysis of upregulated or downregulated pathways in the *RASGRP1*^−/−^ versus *WT INS*-GFP^+^ cells. **k,** Diagram shows the list of genes that are consistently upregulated in the *DLK1*^−/−^ versus *WT* and *RASGRP1*^−/−^ versus *WT INS*-GFP^+^ cells.

Finally, we performed bulk RNA-seq and ATAC-seq to identify the potential downstream target genes or pathways of *DLK1* and *RASGRP1*. PCA (RNA-seq: Fig. 7a; ATAC-seq: Fig. 7b) and clustering analysis (RNA-seq: Extended Data Fig.7a; ATAC-seq: Extended Data Fig.7b) showed that *WT* and *DLK1*^−/−^ hESC-derived INS-GFP^+^ cells clustered separately based on either RNA-seq or ATAC-seq. Further analysis of ATAC-seq data reveals that more than 75% of differentially accessible chromatin regions between *WT* and *DLK1*^−/−^ were located in the promoter region (Fig. 7c). The chromosome is more open in *DLK1*^−/−^ than *WT* hESC-derived INS-GFP^+^ cells (Fig. 7d). IPA analysis with genes (up or down-regulated) and regions (open or closed chromatins) commonly regulated in both RNA-seq and ATAC-seq identified the upregulation of cell stress associated pathways, including EIF2, oxidative phosphorylation, mitochondrial dysfunction, and production of NO and ROS (Fig. 7e).

Similarly, PCA (RNA-seq: Fig. 7f; ATAC-seq: Fig. 7g) and clustering analysis (RNA-seq: Extended Data Fig.7c; ATAC-seq: Extended Data Fig.7d) showed that *WT* and *RASGRP1*^−/−^ hESC-derived INS-GFP^+^ cells clustered separately based on either RNA-seq or ATAC-seq. Further analysis of ATAC-seq data reveals that most differentially accessible chromatin regions between *WT* and *RASGRP1*^−/−^ located in promoter regions and some are in intron regions (Fig. 7h). Different from *DLK1*^−/−^ cells, the chromosome of *RASGRP1*^−/−^ INS-GFP^+^ cells are more closed than *WT* INS-GFP^+^ cells (Fig. 7i). IPA analysis with genes and regions commonly regulated in both RNA-seq and ATAC-seq identified the downregulation of pathways associated with cytoskeleton, such as axonal guidance signaling, reelin signaling in neurons, signaling by Rho Family GTPases, etc (Fig. 7j), suggesting that loss of *RASGRP1* might induce the pancreatic β cell apoptosis by decreasing the stability of cytoskeletons and increase the disassembles of cytoskeletons. Finally, five genes, including *ITGB1, KTI12, TOMD1, PLAG1* and *c7orf50*, were found to be upregulated in both *DLK1*^−/−^ and *RASGRP1*^−/−^ INS-GFP^+^ cells (Fig. 7k). Four of the genes (marked with *) have been identified as related or risk genes for diabetes ^54–57^. In particular, ITGa5/ITGB1 has been found to be the binding target of IL1B. This discovery has shed light on how *DLK1*^−/−^ and *RASGRP1*^−/−^ can make pancreatic β cells more susceptible to T1D or cytokine-induced apoptosis, by increasing the levels of ITGB1.

## Discussion.

Although GWAS have identified many T1D associated loci/genetic variants, the biological functions of these functions/genetic variants are largely unknown. In this study, we reported an integrative pipeline combining single-cell multiomics analysis, GWAS, and isogenic hESCs to prioritize the genes/loci/genetic variants and examine the biological function and mechanism. First, we integrated epigenomic and transcriptomic profiles of human pancreas samples from healthy and AAB^+^ donors to gain a better understanding of how T1D risk variants act across different cell types in the pancreas and cause changes in gene regulation. Our findings indicate that T1D genetic risk variants overlap with active regulatory elements in every pancreatic cell type analyzed, rather than being mediated by only one or a few cell types. These results are consistent with growing evidence linking non-immune cells to mediating T1D risk^3,4,6^. Specifically, our work identifies three loci - *DLK1*/*MEG3, TOX, and RASGRP* - expressed in β cells and other islet cell types as putative causal genes for three independent T1D risk variants. DLK1/MEG3 and TOX loci, mediated through islet endocrine cells, is supported by a previous scATAC-seq study that observed a higher overlap of high-PPA variants in these loci with β-cell regulatory elements^4^. We expand on these findings by predicting *rs1947178* and *rs3783355* as causal variants at these loci and further prioritize *rs55728265* at the *RASGRP1* locus as an additional variant mediating T1D genetic risk through islet endocrine cells.

Next, we employed three types of isogenic hESC systems, including gene knockout, regulatory region knockout and SNP knockin to systematically examine the biological function of the identified genes, regulatory regions, and SNP in human pancreatic β cell generation and survival. Since previous studies have reported the function of LncRNA MEG3 in pancreatic β cells^58^, we decided to focus on three protein coding genes, *DLK1, RASGRP1 or TOX*. Our findings indicate that loss of *DLK1, RASGRP1 or TOX* leads to impaired differentiation toward PP and pancreatic β-like cells, while loss of *DLK1 or RASGRP1* causes increased β cell death. Knockout of the regulatory region of *DLK1 or RASGRP1*leads to the decreased expression of the associated genes, leading to increased β cell death. Furthermore, *rs3783355* risk allele causes decreased *DLK1* expression and increased β cell death. Delta-like non-canonical Notch ligand 1 (DLK1), also known as preadipocyte factor 1 (PREF-1) is a cleavable single-pass transmembrane protein and a member of the Notch/δ/Serrate family. Previous studies of miRNA profiling on T2D islets also identified DLK1-MEG3 miRNA clusters that are downregulated in T2D condition, which further highlighting the important role of DLK1-MEG3 loci in human β cell biology^59^. The overexpression of *DLK1* in pancreatic β cells in mice causes the increased islet mass and insulin secretion^60^. *Dlk1* null mice display a partially penetrant neonatal lethality and a complex pattern of developmental and adult phenotypes. However, the β cell specific *DLK*^−/−^ mice are viable^61^. Here, we found that loss of *DLK* causes the defective pancreatic differentiation and increased β cell death. This discrepancy of mouse and human data highlighting the significance to study T1D associated gene/loci using human cells. RAS guanyl nucleotide-releasing protein (RASGRP) functions as a diacylglycerol DAG-regulated nucleotide exchange factor specifically activating Ras through the exchange of bound GDP for GTP. Although the genetic variants associated with *RASGRP1* have been linked to both T1D and T2D^62,63^, the role of RASGRP1 in human β cells is largely unknown. Here, we found that loss of *RASGRP1*, knockout of the regulatory region of RASGRP1, and risk allele of *rs55728265* leads to increased β cell death. RNA-seq and ATAC-seq suggested that loss of *RASGRP1* leads to the downregulation of cytoskeleton-associated pathway, which is consistent with a previous study that showed RASGRP1 deficiency causes impaired cytoskeletal dynamics in immune cells^64^. Finally, five genes were identified to be upregulated in both *DLK1*^−/−^ and *RASGRP1*^−/−^ INS-GFP^+^ cells, four of which have been identified as related or risk genes for diabetes, which highlights the powerful tools to apply isogenic hESCs to dissect the biological functions and molecular mechanism of T1D associated SNPs.

While the role of immune cells mediating T1D genetic risk is generally understood, it is still unclear how other pancreatic cell types contribute to T1D risk. One hypothesis is that risk variants at these other cell types lead to disease predisposition by promoting the recruitment of self-reactive T-cells or creating a harsher cellular microenvironment that further predisposes β-cell death. Support for this hypothesis is provided by a previous snRNA-seq study from healthy, AAB^+^, and T1D human pancreas, which suggested that T1D ductal cells may help promote CD4^+^ T cell tolerance through the expression of MHC molecules and other surface receptors^8^. Our work indicates that the immune cells indeed have the highest individual contribution to T1D genetic risk. However, this contribution is relatively small compared to all the other cell types combined. In addition to multiple variants acting through islet endocrine cells, we identified a role for acinar, stellate, endothelial, and to a lesser degree, ductal cells as likely mediators of T1D genetic risk. This unexpected finding agrees with and expands on other studies of T1D at the single-cell level identifying the contributions of other pancreatic cell types to T1D genetic risk and onset^4,8^. Therefore, an important question for future studies is understanding how T1D risk variants act through non-immune cell types, particularly β cells.

Although our studies showed the proof of principle to combine the single cell multiomics, GWAS and isogenic hESC lines-derived cells to prioritize and study GWAS identified genes/loci/genetic variants, one limitation is that we jointly analyze pre-diabetic (AAB^+^ without symptomatic presentation) and diabetic donors due to the low sample size. While our results suggest that this is a valid approach to detecting disease-relevant biology, this design would miss molecular signatures associated with different stages of the disease. Therefore, separately studying β cells from T1D donors is an important future direction that can provide essential clues for new therapeutic strategies.

## Methods

### Tissue processing and sample preparation.

Human pancreatic islets were isolated in the Human Islet Core at the University of Pennsylvania following the requirements of the Clinical Islet Transplantation consortium procedure. The pancreatic islets were grown in CIT culture medium and maintained in a humidified incubator with 5% CO2 at 37℃. Single-cell RNA-seq and single-nucleus ATAC-seq were performed using 10X Chromium platform at genomics resources core facility at Weill Cornell Medicine.

### Single-nucleus ATAC-seq processing.

Single-nucleus ATAC-seq data was processed using the Parker Lab snATAC-seq pipeline (https://github.com/porchard/snATACseq-NextFlow). Briefly, after performing adapter trimming with cta (v. 0.12; https://github.com/ParkerLab/cta), reads were aligned to the hg19 reference genome using bwa mem (v. 0.7.15-r1140^65^) using *-I 200,200,5000* to avoid large fragments being artificially assigned low MAPQ scores. Barcode sequences were corrected for sequence mismatches by calculating the Hamming distance between all barcodes and fixing all barcodes with a Hamming distance smaller or equal to 2 to a barcode sequence in the 10X Genomics barcode list. After mapping, we identified barcodes using Picard MarkDuplicates (v. 2.8.1; https://broadinstitute.github.io/picard). We used ataqv (https://github.com/ParkerLab/ataqv^66^) to obtain barcode-level QC metrics, such as the number of high-quality autosomal alignments (HQAA) and transcription start site (TSS) enrichment. For downstream analyses, we retained only barcodes with HQAA ≥ 5,000, TSS enrichment between 3 and 20, and no more than 15% of all reads originating from a single autosome. The latter metric helps to remove barcodes associated with low-integrity nuclei. Doublets were flagged and removed using ArchR (v. 0.9.5)^67^. Because the ambient signal (soup) from the snATAC-seq library is mainly from chrM, which was filtered for our analyses, we did not perform ambient DNA correction. For integration with the scRNA-seq data (described below), we generated count matrices for each library encoding the number of ATAC-seq fragments overlapping promoter (5 Kb upstream of most upstream transcription start site) and gene body regions of autosomal, protein-coding genes using bedtools (v2.26.0).

### Single-cell RNA-seq.

Single-cell RNA-seq data were processed with the Parker Lab snRNA-seq pipeline (https://github.com/porchard/snRNAseq-NextFlow). Reads were aligned to the hg19 reference genome and GENCODE v19^68^ using STARsolo (STAR v. 2.5.4^69^). Barcode sequences were corrected for mismatches using the same approach as in the snATAC-seq data. We then calculated QC metrics for each barcode (number of UMIs, % mitochondrial reads, etc.). We selected for downstream analyses barcodes that had at least 1,000 UMIs and were called non-empty (1% FDR) by EmptyDrops^70^. For each library, we calculated the % mitochondrial reads rank distribution and identified the inflection (knee) using the uik function of the inflection package in R (https://papers.ssrn.com/sol3/papers.cfm?abstract_id=3043076). We only kept barcodes with % mitochondrial reads smaller than the inflection value, ranging from 6.6% to 20.2%. Doublets were flagged and removed using DoubletFinder (v2.0.2)^71^ with default parameters. After removing doublets and barcodes that failed QC, we used DecontX (Celda v1.2.4)^72^ to control for ambient RNA (soup RNAs). We performed a first-pass clustering of the barcodes that passed QC using Seurat (Extended Data Fig. 1) to identify broad cell identities. We then used the first-pass clustering information with DecontX with stringent parameters (delta 1 = 10 and delta 2 = 20) to obtain the ambient-subtracted count matrices for each library. We used the ambient-subtracted count matrices of autosomal, protein-coding genes for downstream analyses.

### Sample genotyping.

Samples were genotyped using the Illumina Infinium 2.5M exome chip (InfiniumOmni2–5Exome-8v1.3_A2). The genotyping call rates for the 16 samples ranged from 99.0% to 99.7%. The SNP probe sequences were remapped to GRCh37 and all problematic SNPs were discarded. This process resulted in a total of 2,522,105 SNPs with genotypes. Next, SNPs that have genotype missingness in >=2 out of our samples and duplicate SNPs with the same genomic coordinates with another one were removed. Further, we merged our genotypes with that of the 1000G phase 3v5 samples^73^. Subsequently, the SNPs with HWE p-value < 1e-4, and palindromic SNPs (A/T, or G/C SNPs) with MAF>0.4 in the merged data set were removed. Phasing was performed on the joint data set of 1,609,033 SNPs using Eagle (v2.4)^74^. Genotypes were imputed using 1000 genomes phase 3 panel in the Michigan Imputation Server using Minimac4 (v1.5.7)^75^ and the 1000G phase 3v5 (GRCh37) reference panel. No sex discrepancy was found by assessing the SNP genotypes using verifybamID^76^ with the reported gender. Sample ICRH135 did not have sufficient DNA for genotyping and was dropped from the genetic analyses.

### CVB4-hg19 alignments.

In order to quantify CVB4 infection efficiency, we aligned scRNA-seq and snATAC-seq reads to a hybrid hg19-CVB4 genome, where the CVB4 genome (GenBank AF311939.1) is appended to hg19 as a separate chromosome. Similarly, we built a hybrid GTF file with the human genes and the CVB4 genome as an additional gene. We generated STAR and bwa indices for the hybrid hg19-cvb4 genome and mapped reads using the same pipeline described below. To quantify the CVB4 infection efficiency, we counted the fraction of reads mapping to the CVB4 portion of the hybrid genome. To independently confirm that our pipeline worked as expected, we used SANDY (https://github.com/galantelab/sandy) to generate hybrid paired-end reads from both genomes using the command *sandy genome* with flag *-- id=” %i.%U read=%c:%t-%n mate=%c:%T-%N length=%r”* and verified that the snATAC-seq and scRNA-seq pipelines aligned these simulated reads to the correct coordinates on both assemblies.

### Cross-modality integration of snATAC-seq and scRNA-seq profiles.

In order to integrate all 34 libraries, we used Seurat (v.4.0.3)^31^. After exhaustively testing different pipelines, we obtained the best results for this dataset using Seurat’s standard workflow. After running the principal component analysis (PCA) step, we extracted the first 30 PC embeddings for each barcode and calculated the Spearman correlation with technical variables (sequencing depth, % mitochondrial reads, etc.) to identify PCs driven by technical aspects. We used PCs 1,3–30 for the FindNeighbors and RunUMAP steps because PC 2 was correlated with sequencing depth. We used options resolution=1, algorithm=2, n.start=1000, and n.iter=1000 for FindClusters and parameters n.neighbors=50 and n.epochs=500 for RunUMAP. This approach yielded 30 clusters in the integrated data. We next identified and removed clusters that could not be unambiguously assigned to any cell type (*i.e.,* loaded on more than one cell-type-specific marker) or had aberrant QC metrics. After filtering these low-quality barcodes, we iteratively merged the remaining clusters based on similar gene express/accessibility patterns to obtain the final cluster assignments used in this study. A subset of the snATAC barcodes assigned to the UMAP region corresponding to the acinar cells could not be unambiguously classified as acinar cells and was removed. This resulted in a higher fraction of scRNA-seq barcodes in the acinar cluster compared to the other clusters. Despite the relatively smaller fraction of acinar snATAC-seq barcodes, the number of barcodes was still higher than most clusters and, therefore, did not substantially affect our chromatin accessibility analyses for the acinar cells.

### Peak calling.

We generated BAM files for each cluster by combining data from all barcodes in that cluster (pseudo-bulk analyses). We also generated BAM files for each cluster/library combination. We used MACS2 (v. 2.1.1.20160309) to call summits on each cluster bam file, and we extended each summit by 150 bp in both directions. The set of extended summits called on the cluster-level bam file (all libraries combined) was labeled as the primary summit list. We assessed the reproducibility of each extended summit in the primary list using bedtools intersect (v2.26.0) to count the number of intersections in the per-library extended summits. We retained for downstream analyses the extended summits from the primary list that 1) overlapped extended summits from at least two different libraries and 2) did not overlap any regions with known mappability issues.

### Differential gene expression analyses.

For each cell type, we tested for association of gene expression with cytokine treatment and CVB4 treatment using DESeq2 v1.34.0^77^ and a pseudo-bulk approach. We filtered lowly expressed genes (DecontX-corrected counts ≥ 1 in ≤ 5 cells across all samples and cell types) using the pp.filter_genes function with min_cells=5 from scanpy v1.5.1^78^, retaining 16,871 genes. To generate the pseudo-bulk count matrix, for each gene, we summed the DecontX-corrected counts across cells within each sample and cell type. Using the rounded pseudo-bulk matrix as input, we modeled the gene expression for each cell type using DESeq2’s *DESeq* function with default options except type=‘LRT’ and sfType=‘poscounts’. We included condition status (i.e., cytokine treated or CVB4 treated), donor ID, sex, age, body mass index (BMI), proportion of donor cells identified as alpha cells (which is a proxy of islet content and accounts for any differences in background RNA persisting after DecontX correction; Extended Data Fig. 2), and mean cell complexity (the average of number of genes detected per cell within each sample^79,80^) as fixed effect covariates. Age, BMI, alpha cell proportion, and cell complexity were standardized to unit variance (mean-centered and scaled). For each model, we performed the likelihood ratio test (LRT) to test for association between gene expression and condition status. Finally, we controlled for the number of tests performed across all cell types using the Benjamini-Hochberg (BH) procedure^81^ and LRT-derived p-values.

### Gene set enrichment.

Gene sets enriched in differentially expressed genes in beta cells. We performed gene set enrichment in differentially expressed genes (FDR<10%) in beta cells across the cytokine and CVB4 treatment in the present study, as well as AAB^+^ status from a larger, more well-powered study^33^. We tested for enriched gene sets from the Gene Ontology (GO) biological processes gene set database^82,83^ using the *compareCluster* function from clusterProfiler v4.2.2^84^ with OrgDb = org.Hs.eg.db::org.Hs.eg.db, ont=‘BP’, and the rest as default parameters. To simplify results and identify the broader biological processes enriched in each condition, we used the R package rrvgo v1.9.1 to collapse redundancy in GO terms. We generated a similarity matrix across all GO terms nominally significant (p < 0.05) in at least one comparison using the calculateSimMatrix function. We then reduced the significant terms for each analysis using the reduceSimMatrixfunction with a threshold parameter of 0.95. For each group of terms under a parent term, we reported the p-value of the most significant term.

Gene sets enriched in differentially expressed genes in other cell types. We also tested for enriched gene sets in the full differential expression results (described in Differential gene expression analyses) for each cell type. We used the fgseaMultilevel function from fGSEA v1.20.0^85^ with eps=0, scoreType=‘std’, and the rest as default parameters. We used the LRT statistic weighted by the direction of the log_2_(fold changes (FCs)) from the DESeq2 results (see Differential gene expression analyses) to pre-rank the genes. We tested gene sets found in the following databases, which were downloaded via the molecular signatures database (MSigDB) v2023.1^86,87^: Kyoto Encyclopedia of Genes and Genomes (KEGG) pathways^88^, BioCarta pathways, and GO biological processes (January 2023 release)^82,83^. This approach is more well-powered than the clusterProfiler approach described above for beta cells as it leverages the full statistics of the DE analyses. However, full summary statistics were not available for the larger AAB+ study^33^, precluding the use of clusterProfiler.

For both approaches, we controlled for the number of tests performed per cell type using the BH procedure.

### Transcription factor binding prediction and chromatin information analyses.

We used BMO and our previously described chromatin information analysis pipeline^34^ available at https://github.com/ParkerLab/BMO/tree/pre-1.1 to predict bound TF motifs and estimate the impact of TFs in their local chromatin architecture. Briefly, we used the hg19 motif scans from a non-redundant position weight matrices collection corresponding to 540 TF motifs^34^. For each cell type pseudo-bulk snATAC-seq BAM file, we calculated the distribution of ATAC-seq fragments overlapping each TF motif instance and the number of co-occurring motifs from the same TF motif within 100 bp to use as input for BMO. BMO predicts TFs using a simple premise that highly accessible motif clusters will be more likely bound by TFs, as the vast majority of TFs cannot induce open chromatin based on DNA sequence alone^34^. BMO fits two negative binomial distributions for the ATAC-seq signal and the number of co-occurring motifs per motif instance and calculates the probability of a given motif instance being bound based on the combined p-value for these two distributions.

Chromatin information for each TF motif was estimated using the feature V-plot information content enrichment (f-VICE) score described in our previous study^34^. Briefly, we generated V-plots (aggregate ATAC-seq fragment midpoint distributions around TF binding sites) for non-overlapping (within 500 bp) BMO-predicted bound instances of a given TF motif (Fig. 3b, top plots). We then calculated the chromatin information (f-VICE score) for each motif by quantifying the log_2_ information content enrichment at TF-adjacent (−25 to +25 from motif) and TF-proximal (−70 to −50 and 50 to 70 bp from motif) regions compared to a randomly shuffled ATAC-seq midpoint distribution (Fig. 3b, bottom signal tracks). These regions are expected to have high information content when the TF induces nucleosome phasing. We then normalized f-VICE scores for each cell type by calculating the residuals of the linear model f-VICE ~ log_10_(total fragments) + log_10_(total co-occurring motifs), which controls for the abundance and overall accessibility of the predicted bound instances for each TF motif.

In order to compare chromatin information across conditions (Fig. 3d), we calculated the f-VICE scores separately for the pseudo-bulk snATAC-seq BAM files obtained from each cell type and donor combination (*i.e.,* Donor 1 β cells, Donor 2 β cells, etc.). First, we calculated f-VICEs separately per donor and cell type to avoid confounding by the different number of nuclei. We then converted each donor and cell type normalized f-VICE distribution into Z-scores. Finally, we calculated the median Z-score for each TF motif to obtain a single value for a TF motif per condition and cell type. For visualizing this data in Fig. 3c, d heatmaps, we calculated row-wise (per motif) Z-scores.

### Differential accessibility analyses.

We used DESeq2 (1.3.2) to perform differential accessibility analyses. We used as input the pseudo-bulk counts from each library for the reproducible extended summits called on each cluster. For the AAB+ versus healthy comparisons, we controlled for age, sex, BMI, median TSS enrichment, and log_10_(HQAA). We scaled and centered age and BMI. For the CVB4 and cytokine versus control comparisons, we opted for a paired design that accounted for donor ID and median TSS enrichment per library, but not age and BMI due to collinearity. Because of statistical instability observed in single-cell approaches for differential analyses in this dataset, we designed an alternative approach to calculate significance based on effect sizes. For each comparison, we removed features with a mean number of reads < 3 and divided the remaining features into 50 equally spaced bins of mean chromatin accessibility using the chop_evenly function from the Santoku R package (https://github.com/hughjonesd/santoku). We removed regions with log_2_ fold-change > 10, as these likely represented technical artifacts from low ATAC-seq coverage. For each of the 50 chromatin accessibility bins, we identified the features in the 80^th^, 85^th^, 90^th^, 95^th^, and 99^th^ percentiles of absolute log_2_ fold-change, which were used for the fGWAS enrichments described below. A summary of this approach is included in Extended Data Fig. 2d.

### Co-accessibility analyses.

Co-accessibility between accessible regions were calculated for each cell type separately by condition using CICERO^50^ with default parameters. We generated count matrices for each pseudo-bulk BAM file representing a cell type and condition (e.g. healthy β cells) for the accessible regions of that cell type (reproducible extended summits, described above). We used as input for CICERO the count matrix and the corresponding UMAP coordinates of each barcode. We annotated the resulting connections based on whether each connected peak overlapped a T1D credible set SNP or a gene TSS from GENCODE V19.

### GWAS enrichments and functional fine-mapping using fGWAS.

We calculated GWAS enrichments in features of interest using fGWAS (commit 0b6533d)^44^. For the GWAS enrichments of the accessible regions per cluster, we ran fGWAS with the *-print* flag using as input the summary statistics from each GWAS study and a reproducible list of extended summits per cluster. For the DARs T1D GWAS enrichments, we used similar steps as above. However, instead of splitting the genome into windows of 5,000 variants based on their order of occurrence (fGWAS default), we generated a bed file of custom 5,000 variant windows where the window corresponding to each T1D loci was centered on the lead variant of the primary signal using the flag *-bed*. The remaining genomic windows were either left unchanged or shortened in case they overlapped a T1D locus chunk. This step was necessary due to the sparseness of the genomic territory covered by DARs. For the functional fine-mapping, we assigned a 0 or 1 value for each T1D variant encoding whether they overlapped a reproducible extended summit in each cell type. We ran fGWAS using the option *-fine* and including all clusters with significant enrichment in the T1D GWAS.

### PPA-weighted chromatin accessibility Z-scores.

To identify which cell types likely mediate T1D genetic risk in each locus, we developed an approach based on the chromatin accessibility for each cell type at the locus. First, we extended each variant in the genetic fine-mapping credible sets (calculated by Chiou *et al.*) by 50 bp in each direction. Next, we counted how many snATAC-seq reads overlapped the extended variant region in the pseudo-bulk data from each cell type. We then normalized the snATAC-seq signal by the sequencing depth and multiplied it by the genetic fine-mapping PPA. When two or more variants overlapped in the extended region, we calculated the ATAC-seq signal for the merged region and used the highest PPA. We retained for analysis only loci where at least one credible set variant overlapped a reproducible (minimum of 2 samples) ATAC-seq broad peak. We then summed each locus’s PPA-weighted chromatin accessibility values to obtain a single score per cell type. Finally, we applied a Z-score transformation for each locus across cell types.

### GWAS variants regulatory impact prediction.

We used LS-GKM ^89^ to train a predictive model of 11-mers for each cell type using as positive regions the extended summits. We used the genNullSeqs function from the gkmSVM R package^90^ to obtain the negative set of GC- and repeat-content matched regions per cell type. To predict the regulatory impact of the SNPs of interest, we used GkmExplain^53^ using as input the ±25 bp flanking each allele and calculated the predicted importance scores for each base. In order to validate the LS-GKM model, we separately calculated the ATAC-seq allelic imbalance at heterozygous SNPs and compared it to the Delta-SVM scores for each allele. Using the genotype data from each donor, we used WASP (v. 0.2.1, commit 5a52185; python version 2.7) ^91^ to diminish reference bias using the same mapping and filtering parameters described for the initial mapping and filtering. Duplicates were removed using WASP’s rmdup_pe.py script. To avoid double-counting alleles, overlapping read pairs were clipped using bamUtil clipOverlap (v. 1.0.14; http://genome.sph.umich.edu/wiki/BamUtil:_clipOverlap). We counted the number of reads containing each allele for each heterozygous autosomal SNP, using only bases with a base quality of at least 10. We further split each donor’s BAM file per cell type to calculate allelic imbalance per cell type separately and for the entire library. We used a two-tailed binomial test that accounted for reference allele bias to evaluate the significance of the allelic bias at each SNP. The observed allelic bias was then correlated with the Delta-SVM score, which was obtained by scoring the 11-mers centered on the REF and ALT alleles for the 1,000 Genomes (Phase 3). We used all SNPs with an absolute Delta-SVM score ≥ 2 to compare with the observed allelic imbalance.

### Genome visualizations.

We used pyGenomeTracks (version 3.7)^92^ to generate genome visualizations of snATAC-seq signals, co-accessible regions, and GWAS variants.

### Maintenance of hESCs.

*INS*^*GFP/W*^ MEL-1 hESCs were grown on Matrigel-coated plates in StemFlex medium (Thermo Fisher), supplemented with 50 µg/mL normocin (InvivoGen). The cells were maintained at 37°C with 5% CO_2_, and were passaged every 4–6 days at a ratio of 1:13 with RelesR (STEM CELL Technologies). All lines were regularly tested for mycoplasma contamination, and all hESC studies were approved by the Tri-Institutional Embryonic Stem Cell Research Committee (ESCRO).

### Stepwise Differentiation.

*WT* and isogenic *INS*^*GFP/W*^ MEL-1 cells were cultured on Matrigel-coated 6-well plates in StemFlex medium (Thermo Fisher) and maintained at 37℃ with 5% CO_2_. hESCs were differentiated using a previously reported strategy^48^. On day 0, cells were exposed to basal medium RPMI 1640 (Corning) supplemented with 1× glutamax (Thermo Fisher), 50 μg/mL normocin, 100 ng/mL Activin A (R&D systems), and 3 μM of CHIR99021 (Cayman Chemical) for 24 hours. The medium was changed on day 2 to basal RPMI 1640 medium supplemented with 1× glutamax, 50 μg/mL normocin, 0.2% FBS (Corning), 100 ng/mL Activin A for 2 days. On day 4, the resulting definitive endoderm cells were cultured in MCDB131 medium supplemented with 1.5 g/L sodium bicarbonate, 1× glutamax, 10 mM glucose, 2% BSA, 50 ng/ml FGF7, 0.25 mM ascorbic acid for 2 days. On day 6, the cells were differentiated in MCDB131 medium supplemented with 2.5 g/L sodium bicarbonate, 1× glutamax, 10 mM glucose, 2% BSA, 0.25 mM ascorbic acid, 2 μM retinoic acid, 0.25 μM SANT1, 50 ng/ml FGF7, 200 nM TPB, 200 nM LDN and 0.5× ITS-X supplement for 2 days. On day 8, the cells were induced to differentiate to pancreatic progenitor stage 2 cells in MCDB131 medium supplemented with 2.5 g/L sodium bicarbonate, 1× glutamax, 10 mM glucose, 2% BSA, 0.25 mM ascorbic acid, 0.2 μM retinoic acid, 0.25 μM SANT1, 2 ng/ml FGF7, 100 nM TPB, 400 nM LDN and 0.5× ITS-X supplement for 3 days. On day 11, the cells were induced to differentiate to insulin expressing cells in MCDB131 medium supplemented with 1.5 g/L sodium bicarbonate, 1× glutamax, 20 mM glucose, 2% BSA, 0.1 μM retinoic acid, 0.25 μM SANT1, 200 nM LDN, 1 μM T3, 10 μM ALKi5, 10 μM zinc sulfate, 10 μg/mL heparin and 0.5× ITS-X for 3 days. On day 14, the cells were further maturated in MCDB131 medium supplemented with 1.5 g/L sodium bicarbonate, 1× glutamax, 20 mM glucose, 2% BSA, 100 nM LDN, 1 μM T3, 10 μM zinc sulfate, 10 μg/mL heparin, 100 nM GS inh XX and 0.5× ITS-X for 9 days for apoptosis analysis. For apoptosis analysis, cells were harvest on day 23.

### Generation of isogenic *DLK1*^−/−^*, RASGRP1*^−/−^*, TOX*^−/−^*, DLK1*^*Δ*^*, RASGRP*^*Δ*^, and *rs3783355*^*A/A*^ hPSC lines.

To create *DLK1*^−/−^*, RASGRP1*^−/−^ and *TOX*^−/−^ hESC lines, three sgRNA targeting exons of *DLK1, RASGRP1* and *TOX* were designed and cloned into a vector carrying a CRISPR-Cas9 gene with puromycin gene (Addgene plasmid #42230). The sgRNAs were validated using the surveyor assay in 293T cells. The constructs containing validated sgRNAs were electroporated into dissociated INS^*GFP/W*^ MEL-1 cells suspended in Human Stem Cell Nucleofector solution (Lonza) following the manufacturer’s instructions. After replating, the electroporated cells were selected with 500 ng/mL puromycin. After 2 days of puromycin selection, hESCs were dissociated by Accutase (Innovative Cell Technologies) and replated at single cells. The single cell culture was supplemented with 10 µM Y-27632 for the first two days. After approximately 10 days, individual colonies were picked, mechanically disaggregated, and replated into two individual wells of 96-well plates. A portion of the cells was lysed and analyzed by Sanger sequencing. *DLK1*^−/−^*, RASGRP1*^−/−^ and *TOX*^−/−^ hESC lines were confirmed by Sanger sequencing. *WT* clonal lines from the targeting experiment were included as *WT* controls to account for potential non-specific effects associated with the gene-targeting process.

To create *DLK1*^*Δ*^ and *RASGRP1*^*Δ*^ hESC lines, four sgRNAs targeting the upstream and downstream of the targeted regions were designed and cloned into a vector carrying a CRISPR-Cas9 gene (Addgene plasmid #42230). The constructs containing validated sgRNAs were electroporated into dissociated INS^*GFP/W*^ MEL-1 cells. After puromycin selection and subcloning as described above, *DLK1*^*Δ*^ and *RASGRP1*^*Δ*^ hESC lines were confirmed by Sanger sequencing. *WT* clonal lines from the targeting experiment were included as *WT* controls to account for potential non-specific effects associated with the gene-targeting process.

To create *rs3783355*^*A/A*^ hESC lines, one sgRNA targeting the regions close to SNP *rs3783355* was designed and cloned into a vector carrying CRISPR-Cas9 gene (Addgene plasmid #42230). The construct containing validated sgRNA with puromycin gene and the mutant template to convert G to A, were co-electroporated into the dissociated INS^*GFP/W*^ MEL-1 cells. After puromycin selection and subcloning as described above, *rs3783355*^*G/G*^ clones from the same targeting experiment were included as controls.

### Immunocytochemistry analysis.

Cells were fixed in 4% paraformaldehyde solution (Affymetrix) for 20 mins, then washed three times in PBS with 5 mins incubation each. The cells were blocked and permeabilized in PBS solution containing 5% horse serum and 0.3% Triton for 1 hour at room temperature. The cells were incubated with primary antibodies overnight at 4°C, followed by three times wash in PBS with 5 mins incubation each. After 1 hour incubation with fluorescence-conjugated secondary antibodies (Alexafluor, ThermoFisher Scientific) at RT, cells were washed with PBS for three times and imaged with LSM 800 confocal microscope (Zeiss). The primary antibodies used were anti-SOX2, anti-OCT4 (1:500–1:1000 according to manufacture instructions, Cell signaling), anti-insulin (1:500, DAKO), and anti-cleaved caspase-3 (1:1000, BD Biosciences). The detailed antibody information has been included as Supplemental Table 10.

### Flow cytometry and intracellular FACS analysis.

hESC-derived cells were dissociated using Accutase. To analyze GFP expression, the cells were resuspended in PBS and used directly for analysis.

For intracellular staining, the cells were fixed and permeabilized using Fixation/Permeabilization Solution Kit (BD Biosciences) according to the manufacturer’s instructions. Briefly, cells were first fixed with fixation/permeabilization buffer for 30 mins at 4°C in the dark and then washed twice with washing buffer with 10 mins incubation each time at room temperature. The fixed cells were incubated with primary antibody overnight at 4°C, washed twice with washing buffer with 10 mins incubation each time at RT. After 30 mins incubation with fluorescence-conjugated secondary antibody at 4°C, cells were washed twice with washing buffer with 10 mins incubation each time at room temperature and re-suspended in PBS buffer for analysis. The following primary antibodies were used: anti-FOXA2, (1:500, Millipore), anti-SOX17 (1:500, R&D) and anti-PDX1 (1:500, R&D). The detailed antibody information was included in Supplemental table 10. Samples were analyzed with an Accuri C6 flow cytometry instrument and the data were processed using Flowjo v10 software.

### Annexin V cellular apoptosis analysis.

hESC-derived cells were dissociated by Accutase and washed with cold PBS. The cells were then stained with the APC/Annexin V apoptosis detection Kit (BD Bioscience, 550474) according to manufacturer’s instructions, the samples were the analyzed by Attune NxT Flow Cytometer (Thermo Fisher) within 30 mins.

### RNA-seq.

Sample QC analysis, cDNA library synthesis, and RNA sequencing were carried out by the Weill Cornell Genomics Core. In brief, the quality of RNA samples was examined by Agilent bioanalyzer (Agilent). cDNA libraries were generated using TruSeq RNA Sample Preparation (Illumina). Each library was sequenced using paired-end 51bp reads on the NovaSeq6000 (Illumina). The sequencing reads were cleaned by trimming adapter sequences and low-quality bases using cutadapt v3.5, and were aligned to the human reference genome (GRCh37) using STAR v2.7.9a. Read counts per gene were extracted using HTSeq-count v0.13.5. Differential expression analysis was performed using R DESeq2 package v1.26.0. The counts data were subjected to a regularized logarithm transformation using the rlog function within the DESeq2 package. The transformed data were utilized to perform a principle component analysis (PCA) using the plotPCA function within the DESeq2 package. Additionally, an unsupervised hierarchical clustering on samples was conducted using the Euclidean distance metric, and the R pheatmap package v1.0.12 was employed to visualize the clustering result.

### ATAC-Seq.

Samples are prepared according to Weill Cornell Medicine Epigenetics Core facility protocol. In brief, 50,000 cells are sorted in Weill Cornell Medicine Flow Cytometry Core Facility. Then cells were washed with 1000 μl of ice-cold PBS and resuspend the pellet in 25 μl of ice cold 1X ATAC Buffer [20mM Tris-HCl (pH 7.4), 20mM NaCl and 6mM MgCl2]. Incubate for 5 min on ice. Add 25 μl of ice cold ATAC-Detergent-buffer [20mM Tris-HCl (pH 7.4), 20mM NaCl and 6mM MgCl2, 0.2% Igepal CA-630, 0.2% Tween 20 and 0.02% Digitonin]. Mix throughout well. Incubate the samples on ice for another 3 min. Then samples are centrifuged and pellets are collected. Resuspend the pellet in the following transposase mixture (Per reaction): 25 μl 2X TD Buffer (Illumina 15027866), 2.5 μl TDE1 (Illumina 15027865), 16.5 ul PBS, 0.5 ul 1% Digitonin, 0.5 ul, 10% Tween-20 and 5 ul H_2_O. Incubate the reaction at 37°C for 30 min in thermomixer (Benchmark) set to 500 rpm. Add 250 uls of Zymo DNA binding buffer to samples (5-fold). Tagmented DNA are purified with Zymo DNA clean and concentrator (Zymo research) according to manufacture instruction. Then samples are submitted to Weill Cornell Medicine Epigenetics Core facility for library preparation and sequencing with paired-end 51 bps on the NovaSeq6000. The sequencing reads underwent a preprocessing step including adapter sequence and low-quality base trimming using cutadapt v3.4. The trimmed reads were aligned to the human GRCh37 reference genome using Bowtie2 v2.4.4. with the parameters -X 2000 --very-sensitive -k 5. Duplicate reads were discarded using Picard v2.26.2. Genrich v0.6.1 was utilized to identify peaks in each replicate sample with the parameters -j -q 0.05 -a 200.0, -e to remove mitochondrial genome and regions not assembled into chromosomes, and -E to exclude ‘N’ homopolymers or high mappability regions in the genome. The identified peaks were loaded into the R DiffBind package v3.2.1 for downstream differential binding analysis. Briefly, consensus peaks were determined for the *WT* and *DLK1−/−* conditions, as well as for the *WT* and *RASGRP1−/−* conditions, by combining peaks that overlapped in at least two replicate samples within each condition. The resulting consensus peak set was generated by taking a union of peaks from both conditions and filtering peaks located in the ENCODE blacklisted regions. Counts of reads overlapping the consensus peak set were calculated for each sample and background normalization was applied. The sample-to-sample correlation heatmap plot was generated using the plotHeatmap function within the DiffBind package. The PCA plot was generated using the plotPCA function within the DiffBind package. Differential binding sites between *WT* and *DLK1−/−* conditions and between *WT* and *RASGRP1−/−* conditions were identified with false detection rate (FDR) < 0.05. Annotation of the differential binding sites were performed using the annotatePeak function, and visualization was achieved in the form of a pie chart using the plotAnnoPie function from the R ChIPseeker package v.1.34.1. A profile heatmap plot was generated using the plotProfile function within the DiffBind package to illustrate differential binding site with FDR < 0.01 and absolute log2 fold change > 0.5.

To identify potential common downstream targets regulated by DLK1 and RASGRP1, we incorporated RNA-seq and ATAC-seq data. Specifically, we screened for genes that either exhibited increased chromatin accessibility and gene expression or decreased chromatin accessibility and gene expression in *DLK1−/−* and *RASGRP1−/−* conditions, respectively, compared to their corresponding *WT.* Subsequently, we selected the intersection of genes from the *DLK1−/−* and *RASGRP1−/−* conditions.

### Western blot analysis.

Whole-cell lysates were generated by scraping cells in cold PBS, and re-suspending in RIPA buffer with Thermo Scientific HALT protease inhibitor cocktail (1:100). Lysates were loaded onto 10% NuPage Bis-Tris gels (Invitrogen), resolved by electrophoresis, and transferred to PVDF membranes (Bio-Rad). Membranes were blocked with 3% bovine serum albumin in TBS + 0.05% Tween for 30 mins and then probed overnight with primary antibody. The antibodies were mouse anti-DLK1 (1:100, Santa cruz), mouse anti-RASGRP (1:100, Santa cruz), rabbit anti-TOX (1:1000, cell signaling) and rabbit anti-GAPDH (1:1000, Cell Signaling). Membranes were washed and incubated for 1 h with HRP anti-rabbit/mouse IgG secondary antibody (1:5000, Bio-rad) in 3% milk-TBS-0.05% Tween and picture were taken with Azure biosystem C600.

### Statistical analysis.

Data are presented as mean±SEM derived from at least three independent biological replicates. Data on biological replicates (*n*) is described in the Fig. legends. All statistical analysis in this paper is two-way Anova. Statistical analysis was performed using GraphPad Prism 8 software. *P* values reflect genotype effect in two-way Anova analysis and were *P<0.05, **P<0.01, ***P<0.001 and ****P<0.0001.

### GWAS data.

T1D summary statistics were downloaded from the EBI Catalog (accession number GCST90012879)

## Extended Data

## Supplementary Material

Supplement 1
