# Supplementary material for: An integrative single-cell multi-omics profiling of human pancreatic islets identifies T1D associated genes and regulatory signals
Source: Res Sq. 2023 Oct 18:rs.3.rs-3343318. Preprint. [Version 1] doi: 10.21203/rs.3.rs-3343318/v1 (PMC10602166; doi:10.21203/rs.3.rs-3343318/v1)
Supplement: Supplement 1 [file NIHPPrs3343318v1-supplement-1.pdf]

Supplemental Tables.

Table 1. Sample metadata.

Sample ID	ID	Age	Sex	Condition	Condition_detailed	BMI
HPAP036	Healthy_5	23	F	NORM	NORM	16
HPAP038	AAB+_1	13	M	T1D	preT1D	18.3
HPAP039	Healthy_7	5	F	NORM	NORM	16.3
HPAP040	Healthy_4	35	M	NORM	NORM	23.98
HPAP044	Healthy_8	3	F	NORM	NORM	12
HPAP045	AAB+_2	27	F	T1D	preT1D	26.2
HPAP055	AAB+_3	24	M	T1D	T1D	27.9
ICRH122	Healthy_3	46	F	NORM	NORM	18.2
HPAP059-Mock	Healthy_6-Mock	35	M	mock	mock	37.96
HPAP059-CVB4	Healthy_6-CVB4	35	M	CVB4	CVB4	37.96
HPAP059-Cyto	Healthy_6-Cyto	35	M	Cytokine	Cytokine	37.96
ICRH134-Mock	Healthy_2-Mock	27	M	mock	mock	25.3
ICRH134-Cyto	Healthy_2-CVB4	27	M	Cytokine	Cytokine	25.3
ICRH134-CVB4	Healthy_2-Cyto	27	M	CVB4	CVB4	25.3
ICRH135-Mock	Healthy_1-Mock	52	M	mock	mock	24.5
ICRH135-Cyto	Healthy_1-CVB4	52	M	Cytokine	Cytokine	24.5
ICRH135-CVB4	Healthy_1-Cyto	52	M	CVB4	CVB4	24.5

Table 2. Overview of snATAC and scRNA-seq libraries.

Cell type	ATAC nuclei	RNA nuclei	Both
Acinar	6,646	18,987	**25,633**
α	9,742	11,409	**21,151**
β	6,000	9,577	**15,577**
δ	1,197	1,506	**2,703**
Ductal	19,088	23,108	**42,196**
Endothelial	1,009	928	**1,937**
Gamma	1,273	865	**2,138**
Immune	930	816	**1,746**
Stellate activated	3,032	2,944	**5,976**
Stellate quiescent	980	1,235	**2,215**
**Total**	**49,897**	**71,375**	**121,272**

Table 3. Differentially expressed genes.

Table 4. Chromatin information patterns.

Table 5. Functional fine-mapping.

Table 6. Predicted bound TF motifs are the prioritized loci.

Table 7. The sequences of sgRNAs used for gene targeting.

Gene	sgRNA Sequence (5'-3')
DLK1$^{-/-}$	*GTCCTTTCCCGAGTACCCGG*
RASGRP$^{-/-}$	*GTGCAACGGCATCTCCCAGT*
TOX$^{-/-}$	*TGCGCCCGACGCTCCCTGTC*
DLK1$^{\Delta}$ **upstream**	*CAGCGCCTCTGTTGGCACGG*
DLK1$^{\Delta}$ **downstream**	*TCAGAGGCGGGTGCTTTGTT*
RASGRP1$^{\Delta}$ **upstream**	*TCCAGGCATAGGTATCTCAG*
RASGRP1$^{\Delta}$ **downstream**	*CTACACCCACCGACGCCAGG*

Table 8. PCR and sequencing primers used for genotyping the gene knockout, regulatory region knock and SNP knockin hESC lines.

Gene	Primer Sequence (5’-3’)	
DLK1$^{-/-}$ **(PCR)**	**F**	*CCTCTTACTCCAGACCCCAC*
	**R**	*CCCGTGAATACTCCCATCCA*
DLK1$^{-/-}$ **(Sequencing)**	**R**	*GGGTTAGGCTGAAAGGGTCT*
RASGRP$^{-/-}$ **(PCR)**	**F**	*TCCCTCCCATCATGCTTGTT*
	**R**	*AAGCTGGAGGAAAAGGGGAT*
RASGRP$^{-/-}$ **(Sequencing)**	**F**	*AGCCATCAACTGAGCAGACT*
TOX$^{-/-}$ **(PCR)**	**F**	*CACCTCACTCTGTTCCGTCT*
	**R**	*AATCGTGTCACTTTCCGCAC*
TOX$^{-/-}$ **(Sequencing)**	**F**	*GTTCCGTCTAAGCTTGTTTTGC*
DLK1$^{\Delta}$ **(PCR)**	**F**	*TCTGTCGTTTGTTTGCTGGG*
	**R**	*TGATCAGTGCATGGGTGACT*
RASGRP1$^{\Delta}$ **(PCR)**	**F**	*CCGTCCTCTTCCCCTTACAA*
	**R**	*CCAGGCAGCTTTGAGTTTGT*
rs3783355$^{A/A}$ **(PCR+Sequencing)**	**F**	*CCTCACAAAGGTACAGGAAA*
	**R**	*AGAAAGCATTGGTGAACACT*

Table 9. QPCR primers sequence.

Gene	Primer Sequence (5’-3’)	
DLK1	**F**	*CCCTGTGTGATCAACGGCT*
	**R**	*AGGTCTTGTCGATGAAGCCG*
RASGRP1	**F**	*TGGGTGTGCATCTCAAGGAC*
	**R**	*CCGGGCATAGGAAAGCTCAT*
ACTB	**F**	*CAATGTGGCCGAGGACTTTG*
	**R**	*CATTCTCCTTAGAGAGAAGTGG*

Table 10. Antibodies used for immunocytochemistry and/or intracellular flow cytometry analysis.

Usage	Antibody	Clone #	Host	Catalog #	Vendor	Dilution
Immunostaining	Anti-Insulin	Polyclonal	Guinea Pig	#A0564	Dako	1:500
Immunostaining	Anti-Caspase 3	Monoclonal	Rabbit	559565	BD Biosciences	1:1000
Flow cytometry	Anti-PDX1	Polyclonal	Goat	AF2419	R & D Aquatics	1:500
Immunostaining	Anti-SOX2	Monoclonal	Rabbit	3579S	Cell Signaling	1:400
Immunostaining	Anti-OCT4	Monoclonal	Mouse	Sc-5279	Santa Cruz	1:200
Flow cytometry	Anti-FOXA2	Polyclonal	Rabbit	07-633	Millipore	1:500
Flow cytometry	Anti-SOX17	Polyclonal	Goat	AF1924	R & D Systems	1:500
Immunostaining	Alexa Fluor 488 AffiniPure Anti-Guinea Pig IgG (H+L)	Polyclonal	Donkey	#706-545-148	Jackson ImmunoResearch Labs	1:500
Flow cytometry	anti-Goat IgG (H+L) Highly Cross-Adsorbed Secondary Antibody, Alexa Fluor 488	Polyclonal	Donkey	#A-11055	Thermo Fisher Scientific	1:500
Immunostaining	anti-Rabbit IgG (H+L) Highly Cross-Adsorbed Secondary Antibody, Alexa Fluor 594	Polyclonal	Donkey	#A-21207	Thermo Fisher Scientific	1:500

Flow cytometry	anti-Rabbit IgG (H+L) Highly Cross-Adsorbed Secondary Antibody, Alexa Fluor 647	Polyclonal	Donkey	#A-32795	Thermo Fisher Scientific	1:500
Immunostaining/ Flow cytometry	anti-Mouse IgG (H+L) Cross-Adsorbed Secondary Antibody, Alexa Fluor 647	Polyclonal	Donkey	#A-32787	Thermo Fisher Scientific	1:500
Flow Cytometry	APC Annexin V	unknown	unknown	550475	BD Biosciences	1:20
Western blot	Anti-DLK1	Monoclonal	Mouse	Sc-376755	Santa cruz	1:100
Western blot	Anti-RASGRP1	Monoclonal	Mouse	Sc-365358	Santa cruz	1:100
Western blot	Anti-TOX	Monoclonal	Rabbit	E6I3Q	Cell signaling	1:1000

Supplementary Files

This is a list of supplementary files associated with this preprint. Click to download.

- TableS3Differentiallyexpressedgenes.xlsx
- TableS4Chromatininformationpatterns.xlsx
- TableS5Functionalfinemapping.xlsx
- TableS6PredictedboundTFmotifsatprioritizedloci.xlsx
